# Levels, trends, and determinants of cause-of-death diversity in a global perspective: 1990–2019

**DOI:** 10.1186/s12889-023-15502-4

**Published:** 2023-04-05

**Authors:** Júlia Almeida Calazans, Iñaki Permanyer

**Affiliations:** 1 Centre for Demographic Studies, Universitat Autònoma de Barcelona, Bellaterra, Spain; 2 ICREA- Institució Catalana de Recerca I Estudis Avançats, Barcelona, Spain

**Keywords:** Cause of death diversity, Health inequality, Mortality, Fractionalization index, Ageing

## Abstract

**Background:**

While much is known about the leading causes of death (CoD) and how they have evolved over time, much less is known about the *diversity* of such causes of death. CoD diversity is an important marker of population health heterogeneity that has been largely overlooked in the study of contemporary health dynamics.

**Methods:**

We provide regional and national estimates of CoD diversity from 1990 to 2019. We rely on data from the Global Burden of Disease project, using information on 21 CoD. Results are presented for 204 countries and territories, for women and men separately. CoD diversity is measured with the index of Fractionalization. Results are disaggregated by age and cause of death.

**Results:**

CoD diversity has declined across world regions, except for Latin America and the Caribbean, the region of High-income countries and women in Central Europe, Eastern Europe, and Central Asia. Changes in mortality at adult and older ages have been mostly responsible for CoD diversity dynamics, except for the regions of South Asia and Sub-Saharan Africa, where infant and child mortality still play a non-negligible role. The relationship between CoD diversity, life expectancy, and lifespan inequality is strongly non-monotonic, with turning points differing by sex and indicator. Among longevity vanguard countries, further increases in life expectancy are associated with decreasing lifespan inequality but increasing CoD diversity.

**Conclusion:**

As mortality declines, there is no universal pathway toward low CoD diversity, thus casting doubts on the ability of Epidemiological Transition Theory to predict prospective CoD dynamics among high- and middle-mortality countries. Despite the postponement and increasing predictability of the ages at which individuals die, low-mortality populations are composed of an increasingly heterogenous mix of robust and frail individuals, thus increasing the diversity of health profiles among older persons – an issue that could potentially complicate further improvements in longevity.

**Supplementary Information:**

The online version contains supplementary material available at 10.1186/s12889-023-15502-4.

## Background

During the last decades, most countries have experienced significant increases in life expectancy: individuals worldwide can now expect to survive to ages deemed unattainable not long ago [1, 2]. At the same time, longevity increases have gone hand in hand with a compression in the distribution of the ages at which individuals die – thus reducing lifespan inequality and making age-at-death increasingly predictable [3–6]. There have been significant shifts both in the ages at which individuals die, with generalized mortality reductions at all ages, and in the distribution of causes of death (CoD), from a preponderance of communicable deaths to a majority of non-communicable deaths [7–10]. While much is known about the leading causes of death and how they have evolved over time, much less is known about the *diversity* of such causes of death.

CoD diversity is an important marker of heterogeneity in population health, reflecting how societies are structured and how their inhabitants behave. At one end, low diversity implies more homogeneity and greater predictability regarding the drivers of mortality. At the other end, high CoD diversity implies major challenges to healthcare systems, with the efforts for total mortality reduction becoming more complex, fragmented, and possibly less effective [11]. For these reasons, the mortality profile by cause has long been known to be a key indicator of well-being and health [9, 11]. However, surprisingly little has been investigated about CoD diversity and its drivers over time [11–14].

The main aims of this paper are twofold. First, we document the levels, trends, and drivers of CoD diversity across countries around the globe from 1990 to 2019. Second, we explore the relationship between our CoD diversity indicators and traditional measures of population health, like life expectancy (which measures the average number of years individuals are expected to live) and life disparity (which measures the variability in the ages at which individuals die). So far, empirical evidence on these issues is restricted to a small group of low-mortality countries [11], so little can be said a priori about what the results might show on a global scale. On the one hand, since people dying at different ages are more likely to die from various causes, generalized declines in lifespan inequality could lead to declines in CoD diversity. On the other hand, several studies suggest that increasing survivorship might have led to increasing heterogeneity of health profiles among older persons [6, 15], thus potentially increasing CoD diversity. Alternatively, the Epidemiological Transition theory [7, 8] would predict that, as countries transition from a majority of communicable deaths to a majority of non-communicable deaths, CoD diversity should first increase and then decrease (i.e., follow an inverted U trajectory). On top of that, health outcomes are increasingly stratified by socioeconomic status within countries [16–20], a phenomenon that could further contribute to diversifying the causes from which individuals die. The findings reported in this paper contribute to our understanding of how epidemiological transitions evolve across world regions, how they compare vis-à-vis each other, and how they are related to the swift aging process unfolding around the globe.

## Methods

### Data

We use mortality information from the Global Burden of Disease (GBD) project (https://vizhub.healthdata.org/gbd-results/) from the I*nstitute for Health Metrics and Evaluation* (*IHME*). GBD classifies deaths into 21 CoD (level 2), which, in turn, can be grouped into three major categories (level 1): (1) communicable, maternal, neonatal, and nutritional diseases, (2) non-communicable diseases, and (3) injuries. The information is available for 204 countries/territories nested within seven regions: Central Europe, Eastern Europe, and Central Asia (CEC); High-income (HI); Latin America and the Caribbean (LAC); North Africa and Middle East (MENA); South Asia (SA); Southeast Asia, East Asia, and Oceania (SEO); and Sub-Saharan Africa (SSA). The territorial classification of countries can be found in Additional file 1.

### CoD diversity

CoD diversity is measured using the Fractionalization index, $F$ [21]. Assuming all deaths are classified in a list of mutually exclusive causes, $F$ is defined as the probability that two randomly chosen deaths are attributable to different causes (Additional file 2.1) 1. Lower values indicate that deaths are increasingly concentrated in fewer causes. In the limit, if all individuals died from the same cause, $F$ would equal zero. At the other extreme, higher values of $F$ indicate that the causes from which individuals die become increasingly diverse. The $F$ index is maximized when deaths are equally distributed across all possible causes (when this happens, $F$ equals $(k-1)/k$, where $k$ is the number of CoD – which corresponds to $20/21\cong 0.95$ in our setting).

When calculating the $F$ index, we use the proportion of deaths by cause from the corresponding life tables (rather than the observed number of deaths) to render populations with different age structures comparable (see Additional file 3.3 for calculations of the $F$ index without age structure correction).

#### Life expectancy and life disparity

In addition to death probabilities, the GBD project also provides estimates of life expectancy at birth. This indicator measures the average number of years a synthetic cohort would be expected to live if its members were to experience prevailing age-specific mortality rates throughout their lifetimes, from birth to death (Additional file 2.2). The variability in ages at death is measured by the ‘life disparity’ indicator [4] (also known as e-dagger, or $e^{\dagger}$), which measures how much lifespans differ among individuals (Additional file 2.3). Lower (resp. higher) values of $e^{\dagger}$ indicate less (resp. more) variability in the corresponding age-at-death distribution.

To explore the relationship between the CoD diversity with life expectancy and life disparity indicators, we use *locally estimated scatterplot smoothing* (LOESS) curves [22], weighted by population size. This non-parametric technique generates a best-fit smooth curve through a set of data points to describe the relationship between any two cardinal scale variables.

#### Decomposition of CoD diversity change by causes and age groups

We analyze the contribution of each CoD and age group to *changes* in CoD diversity between 1990 and 2019 (i.e., $\Delta F$) applying the Horiuchi decomposition method [23] (Additional file 2.4). Several studies have shown how this method can be applied to decompose changes in many life table functions, like life expectancy, or lifespan variation [24–28].

#### Uncertainty analysis

We assessed the uncertainty of the estimates based on the uncertainty of the input data from GBD. Uncertainty was obtained by sampling from the corresponding uncertainty intervals reported by GBD using Monte Carlo simulation techniques (Additional file 2.5).

## Results

### CoD mortality profiles

**Fig. 1:**
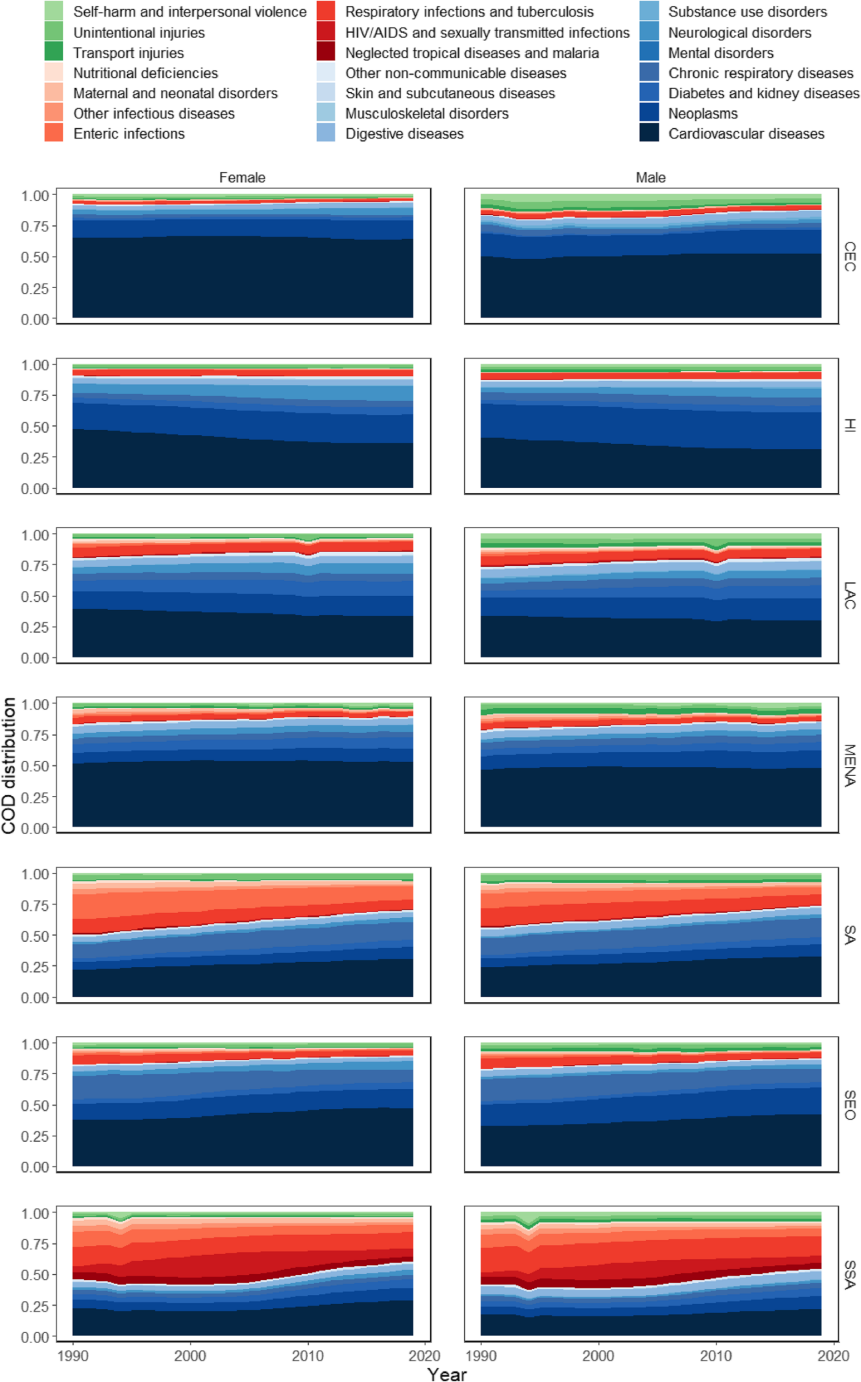
Deaths distribution by cause from life table, by sex and region (1990 – 2019). Source: Global Burden of Disease (GBD/IHME). Note: Central Europe, Eastern Europe, and Central Asia (CEC); High-income (HI); Latin America and the Caribbean (LAC); North Africa and Middle East (MENA); South Asia (SA); Southeast Asia, East Asia, and Oceania (SEO); and Sub-Saharan Africa (SSA)

CoD mortality profiles have been changing smoothly in all world regions, usually without abrupt alterations, between 1990 and 2019 (see Fig. 1 and the 95% uncertainty intervals in Additional file 3.1). Non-communicable deaths were already dominant in 1990 in most world regions (see blue colored shades in Fig. 1) and have become even more prevalent over time. The increase in the share of cardiovascular deaths over total mortality is the main driver for these changes, except for HI, LAC, and women on CEC, where their contribution decreases over time. The share of cardiovascular deaths drops from 41% (95% uncertainty interval 39.74% to 42.21%) to 31% (29.48% to 32.91%) for men and from 48% (45.28% to 51.01%) to 36% (32.88% to 38.87%) for women in HI. The share of cardiovascular deaths falls from 34% (32.17% to 35.65%) to 30% (27.99% to 32.26%) for men and from 39% (36.75% to 41.89%) to 34% (30.72% to 36.55%) for women in LAC. This cause explains around 65% of the total deaths of women in CEC. In addition, neoplasms, neurological disorders, diabetes, and renal diseases increase their relative share in all regions.

The share of communicable, maternal, neonatal, and nutritional deaths over total mortality reduces in most regions (see red colored shades in Fig. 1), with particularly sharp reduction in SA and SSA. The share of those deaths drops from 36% (30.01% to 44.3%) to 19% (15.47% to 23.19%) for men and from 44% (35.13 to 55.43%) to 23% (17.30% to 31.01%) for women in SA and from 50% (41.48% to 59.54%) to 37% (32.06% to 44.02%) for men and from 50% (41.09% to 60.05%) to 36% (30.07% to 42.59%) for women in SSA. Major reductions take place in mortality by maternal and neonatal disorders, neglected tropical diseases, malaria, and other infectious diseases. In contrast, the proportion of those causes remains relatively constant at around 6% in HI countries.

Despite occasional fluctuations due to natural phenomena (e.g., tsunamis and earthquakes) and armed conflicts such as in Rwanda in 1994, the injuries share over total mortality shows a slight reduction over time, particularly for males (see green colored shades in Fig. 1). This reduction is mainly due to the decrease in mortality from self-harm and interpersonal violence in all regions of the world, except for MENA. In this region, the proportion of deaths from self-harm and interpersonal violence increased from 1.27% (1.27% to 1.27%) to 1.82% (1.75% to 1.90%) for males and from 0.52% (0.49% to 0.55%) to 1.17% (1.10% to 1.24%) for females.

While each region has its own peculiarities, the CoD profiles are relatively similar around the globe, except for SSA. Although non-communicable deaths play an important role in this region, especially from 2005 onwards, a considerable portion of the deaths is still due to communicable, maternal, neonatal, and nutritional diseases, especially HIV/AIDS, sexually transmitted infections, respiratory infectious diseases, and tuberculosis. The rise and fall of HIV/AIDS have been particularly pronounced in SSA, thus affecting the mortality profile of that region throughout the entire period.

### Levels and trends in CoD diversity

In 1990, CoD diversity ranged between 0.551 (0.518 to 0.587) in CEC and 0.888 (0.875 to 0.901) in SSA for women, and between 0.702 (0.689 to 0.715) in CEC and 0.897 (0.886 to 0.908) in SSA for men. In 2019, the $F$ index ranged between 0.563 (0.517 to 0.614) in CEC and 0.867 (0.844 to 0.890) in SSA for women and between 0.682 (0.655 to 0.708) in CEC and 0.892 (0.877 to 0.907) in SSA for men (Table 1). Between 1990 and 2019, CoD mortality profiles became more diverse for both sexes in HI and LAC (and women in CEC) and less diverse for the remaining regions. However, the differences over time are only statistically significant for SEO, HI, and SA.

**Table 1:** Fractionalization index, by sex and region (1990 and 2019)

	1990	2019	dif
**Females**			
SEO	0.795 (0.771 to 0.820)	0.732 (0.695 to 0.771)	-0.063^a^
CEC	0.551 (0.518 to 0.587)	0.563 (0.517 to 0.614)	0.012
HI	0.712 (0.687 to 0.739)	0.791 (0.776 to 0.807)	0.079^a^
LAC	0.801 (0.782 to 0.820)	0.827 (0.812 to 0.842)	0.026
MENA	0.712 (0.662 to 0.766)	0.685 (0.640 to 0.732)	-0.027
SA	0.872 (0.871 to 0.872)	0.849 (0.832 to 0.866)	-0.023^a^
SSA	0.888 (0.875 to 0.901)	0.867 (0.844 to 0.890)	-0.021
**Males**			
SEO	0.819 (0.800 to 0.838)	0.757 (0.734 to 0.780)	-0.062^a^
CEC	0.702 (0.689 to 0.715)	0.682 (0.655 to 0.708)	-0.020
HI	0.748 (0.735 to 0.761)	0.796 (0.781 to 0.811)	0.048^a^
LAC	0.840 (0.829 to 0.852)	0.848 (0.835 to 0.860)	0.007
MENA	0.756 (0.715 to 0.798)	0.729 (0.694 to 0.767)	-0.026
SA	0.875 (0.866 to 0.884)	0.839 (0.819 to 0.860)	-0.036^a^
SSA	0.897 (0.886 to 0.908)	0.892 (0.877 to 0.907)	-0.005

Source: Global Burden of Disease (GBD/IHME). Notes: 95% uncertainty interval in parenthesis

*CEC* Central Europe, Eastern Europe, and Central Asia, *HI* High-income, *LAC* Latin America and the Caribbean, *MENA* North Africa and Middle East, *SA* South Asia, *SEO* Southeast Asia, East Asia, and Oceania and *SSA* Sub-Saharan Africa

^a^The difference in the fractionalization index between 1990 and 2019 is statistically significant at a 5% uncertainty level

CoD diversity is generally higher among men than among women across world regions. The largest difference in CoD diversity between men and women is observed in CEC, while SA exhibits the smallest difference. These gender differences are mostly explained by the larger share of injuries mortality among men. As the mortality profile of all regions is dominated by non-communicable diseases, the greater the proportion of deaths from injuries, the greater the CoD diversity. Between 1990 and 2019, the sex gap in CoD diversity remains constant for MENA, it increases in SEO and SSA and it decreases for the other regions. In 2019, the mortality profile by cause becomes slightly more diverse for women than for men in SA.

### Decompositions by cause and age group

Variations in non-communicable deaths are the main determinant of CoD diversity changes between 1990 and 2019 (Tables 2, 3 and the 95% uncertainty interval in Additional file 3.3). More specifically, the increase in the share of cardiovascular deaths led to a decrease in CoD diversity for both sexes in SEO, MENA, SSA, and SA, and for men in CEC. In turn, declines in the share of cardiovascular mortality have led to increases in CoD diversity for both sexes in HI, LAC, and for women in CEC. For instance, changes in cardiovascular mortality alone would have contributed to an increase of 0.071 units in $F$ for males in HI (Table 3).

**Table 2:** Decomposition of the fractionalization index variation, by region – Females (1990 to 2019)

	**SEO**	**CEC**	**HI**	**LAC**	**MENA**	**SA**	**SSA**
**Non-communicable diseases**							
Cardiovascular diseases	-0.082^a^	0.016^a^	0.103^a^	0.042^a^	-0.021^a^	-0.045^a^	-0.034^a^
Neoplasms	-0.007^a^	-0.003^a^	-0.011^a^	-0.005^a^	-0.005^a^	-0.006^a^	-0.005^a^
Diabetes and kidney diseases	-0.002^a^	0.000	-0.002^a^	-0.007^a^	-0.003^a^	-0.003^a^	-0.003^a^
Chronic respiratory diseases	0.025^a^	0.001^a^	-0.002^a^	-0.001^a^	0.000^a^	-0.008^a^	-0.000^a^
Mental disorders	-0.000^a^	-0.000^a^	-0.000^a^	-0.000^a^	0.000	0.000	0.000
Neurological disorders	-0.004	-0.002	-0.009	-0.005	-0.002	-0.001	-0.001
Substance use disorders	0.000^a^	0.000^a^	-0.000^a^	0.000^a^	-0.000^a^	-0.000^a^	-0.000^a^
Digestive diseases	0.001^a^	-0.001^a^	-0.000^a^	-0.000^a^	0.001^a^	0.000^a^	-0.000^a^
Musculoskeletal disorders	-0.000^a^	-0.000^a^	-0.000^a^	-0.000^a^	-0.000^a^	-0.000^a^	-0.000^a^
Skin and subcutaneous diseases	-0.000^a^	-0.000^a^	-0.000^a^	-0.000^a^	-0.000^a^	-0.000^a^	-0.000^a^
Other non-communicable diseases	0.000^a^	0.000^a^	-0.000^a^	-0.000^a^	0.000^a^	-0.000^a^	0.000
**Communicable, maternal, neonatal, and nutritional diseases**							
Neglected tropical diseases and malaria	0.000	0.000	0.000^a^	0.000^a^	0.000	0.000	0.002^a^
HIV/AIDS and sexually transmitted infections	-0.000^a^	-0.000^a^	0.000^a^	0.000^a^	0.000	0.000^a^	-0.002^a^
Respiratory infections and tuberculosis	0.004^a^	0.000^a^	0.000^a^	0.001^a^	0.001^a^	0.009^a^	0.009^a^
Enteric infections	0.000	0.000^a^	-0.000^a^	0.001^a^	0.000^a^	0.028^a^	0.011^a^
Other infectious diseases	0.000^a^	0.000^a^	0.000^a^	0.000^a^	0.000^a^	0.001^a^	0.002^a^
Maternal and neonatal disorders	0.000^a^	0.000^a^	0.000^a^	0.000^a^	0.001^a^	0.002^a^	0.001^a^
Nutritional deficiencies	0.000^a^	0.000^a^	-0.000^a^	0.000^a^	0.000	0.001^a^	0.000^a^
**Injuries**							
Transport injuries	0.000^a^	0.000^a^	0.000^a^	0.000^a^	0.000^a^	-0.000^a^	0.000^a^
Self-harm and interpersonal violence	0.000^a^	0.000^a^	0.000^a^	0.000^a^	-0.000^a^	0.000^a^	0.000^a^
Unintentional injuries	-0.000^a^	0.000^a^	-0.000^a^	-0.000^a^	0.000^a^	-0.001^a^	-0.000^a^
**Total**	**-0.063**	**0.012**	**0.079**	**0.026**	**-0.027**	**-0.023**	**-0.021**

Source: Global Burden of Disease (GBD/IHME)

*CEC* Central Europe, Eastern Europe, and Central Asia, *HI* High-income, *LAC* Latin America and the Caribbean, *MENA* North Africa and Middle East, *SA* South Asia, *SEO* Southeast Asia, East Asia, and Oceania and *SSA* Sub-Saharan Africa

^a^Contribution statistically different from zero at 5% uncertainty level

**Table 3:** Decomposition of the fractionalization index variation, by region – Males (1990 to 2019)

	SEO	CEC	HI	LAC	MENA	SA	SSA
**Non-communicable diseases**							
Cardiovascular diseases	-0.068^a^	-0.021^a^	0.071^a^	0.024^a^	-0.019^a^	-0.049^a^	-0.020^a^
Neoplasms	-0.018^a^	-0.001^a^	-0.016^a^	-0.010^a^	-0.008^a^	-0.005^a^	-0.006^a^
Diabetes and kidney diseases	-0.001^a^	0.000	-0.002^a^	-0.008^a^	-0.002^a^	-0.003^a^	-0.002^a^
Chronic respiratory diseases	0.019^a^	0.002^a^	-0.001^a^	-0.001^a^	0.000	-0.005^a^	-0.000^a^
Mental disorders	-0.000^a^	0.000^a^	0.000^a^	0.000	-0.000^a^	0.000	0.000
Neurological disorders	-0.001	-0.001	-0.004	-0.002	-0.001	-0.001	0.000
Substance use disorders	0.000^a^	-0.000^a^	-0.000^a^	0.000^a^	-0.000^a^	0.000^a^	-0.000^a^
Digestive diseases	0.001^a^	-0.001^a^	0.000^a^	0.000	0.000	0.001^a^	-0.001^a^
Musculoskeletal disorders	-0.000^a^	-0.000^a^	-0.000^a^	-0.000^a^	-0.000^a^	-0.000^a^	-0.000^a^
Skin and subcutaneous diseases	-0.000^a^	-0.000^a^	0.000	-0.000^a^	-0.000^a^	-0.000^a^	-0.000^a^
Other non-communicable diseases	0.000^a^	0.000^a^	-0.000^a^	-0.000^a^	0.000^a^	-0.000^a^	0.000
**Communicable, maternal, neonatal, and nutritional diseases**							
Neglected tropical diseases and malaria	0.000	0.000	0.000	0.000^a^	0.000	0.000	0.002^a^
HIV/AIDS and sexually transmitted infections	-0.000^a^	-0.000^a^	0.000^a^	-0.000^a^	0.000	-0.000^a^	-0.002^a^
Respiratory infections and tuberculosis	0.005^a^	0.000^a^	-0.001^a^	0.002^a^	0.001^a^	0.014^a^	0.015^a^
Enteric infections	0.000^a^	0.000^a^	-0.000^a^	0.001^a^	0.000^a^	0.010^a^	0.007^a^
Other infectious diseases	0.000^a^	0.000^a^	0.000^a^	0.000^a^	0.000^a^	0.001^a^	0.002^a^
Maternal and neonatal disorders	0.000^a^	0.000^a^	0.000^a^	0.000^a^	0.001^a^	0.001^a^	0.000^a^
Nutritional deficiencies	0.000^a^	0.000^a^	-0.000^a^	0.000^a^	0.000	0.000^a^	0.000^a^
**Injuries**							
Transport injuries	0.000^a^	0.000^a^	0.000^a^	0.001^a^	0.001^a^	-0.000^a^	-0.000^a^
Self-harm and interpersonal violence	0.000^a^	0.000^a^	0.000^a^	0.000^a^	-0.000^a^	0.000^a^	0.001^a^
Unintentional injuries	0.000^a^	0.001^a^	-0.000^a^	0.000^a^	0.001^a^	0.000^a^	-0.000^a^
**Total**	**-0.062**	**-0.020**	**0.048**	**0.007**	**-0.026**	**-0.036**	**-0.005**

Source: Global Burden of Disease (GBD/IHME)

*CEC* Central Europe, Eastern Europe, and Central Asia, *HI* High-income, *LAC* Latin America and the Caribbean, *MENA* North Africa and Middle East, *SA* South Asia, *SEO* Southeast Asia, East Asia, and Oceania and *SSA* Sub-Saharan Africa

^a^Contribution statistically different from zero at 5% uncertainty level

The growing share of neoplasms in total mortality contributes to decreasing CoD diversity in all regions. To a lesser extent, the variations in neurological disorders (except for males in SSA) and substance use disorders (except for CEC) also contribute to decreasing CoD diversity in all regions. Changes in *chronic respiratory* disease mortality increase CoD diversity in SEO and CEC and decrease CoD diversity in HI, LAC, and SA. Other non-communicable diseases are causes of death with low shares in both periods and with low variation over time, so their effect on CoD diversity is close to zero.

Mortality reductions in *communicable, maternal, neonatal, and nutritional diseases* increase CoD diversity in LAC, MENA, SA, SEO, and SSA, while the effect is close to zero for the other regions (Tables 2 and 3). More specifically, changes in neglected tropical diseases and malaria mortality increase CoD diversity, while changes in HIV/AIDS and sexually transmitted infections mortality reduce CoD diversity in SSA. Changes in *maternal and neonatal disorders* mortality trigger CoD diversity increases for both sexes in MENA and SA and women in SSA. The remaining communicable diseases contribute very little to changes in CoD diversity. *Injuries* have a minimal effect on the variation of the Fractionalization index over time (Tables 2 and 3).

**Fig. 2:**
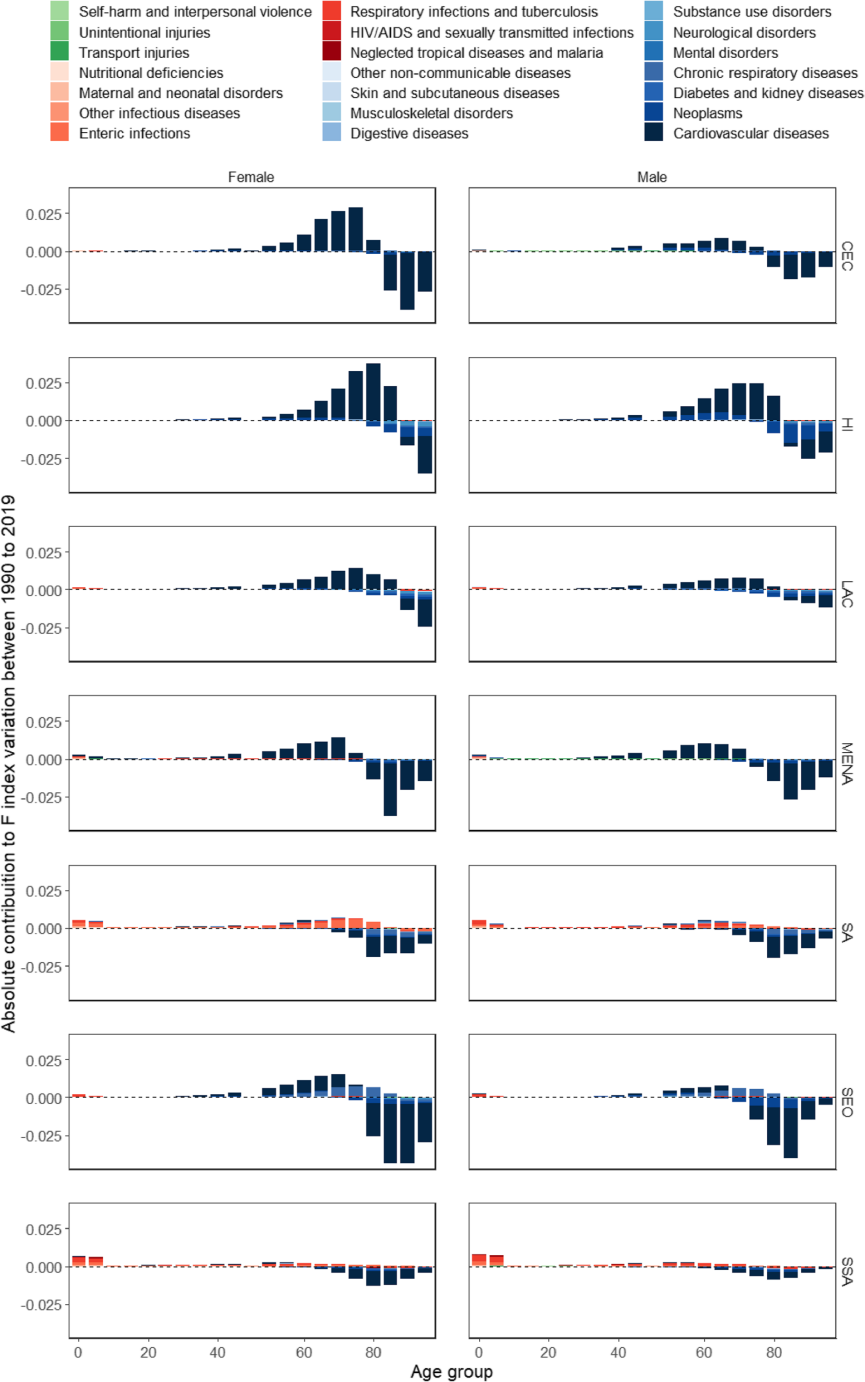
Cause-age decomposition of fractionalization index variation, by sex and region (1990 to 2019). Source: Global Burden of Disease (GBD/IHME). Note: Central Europe, Eastern Europe, and Central Asia (CEC); High-income (HI); Latin America and the Caribbean (LAC); North Africa and Middle East (MENA); South Asia (SA); Southeast Asia, East Asia, and Oceania (SEO); and Sub-Saharan Africa (SSA)

The contribution of cause-specific mortality to CoD diversity changes varies substantially by age (Fig. 2). For non-communicable diseases, mortality changes at young ages have little effect on CoD diversity variation. However, as we move forward in the age distribution, changes in mortality from non-communicable diseases contribute to the increase in CoD diversity. At older ages, beyond a certain threshold that varies between 65 and 85 years old according to region and sex, non-communicable diseases contribute to reducing CoD diversity (see blue shaded bars in Fig. 2).

On the other hand, the variations in communicable, maternal, neonatal, and nutritional diseases mortality contribute to increase CoD diversity over time, even if the effect is much smaller than the one found for non-communicable diseases (see red shaded bars in Fig. 2). In general, the largest contributions are concentrated at younger ages (especially in the 0–1 and 1–5 age groups). However, it is also possible to observe that for SA and SSA, the variations of mortality in adulthood for these causes also contribute non-negligibly to the increase in CoD diversity. Finally, the variations in injuries contribute to a slight increase in diversity and are mainly concentrated at younger ages (see green shaded bars in Fig. 2).

### Relationship with population health measures

**Fig. 3:**
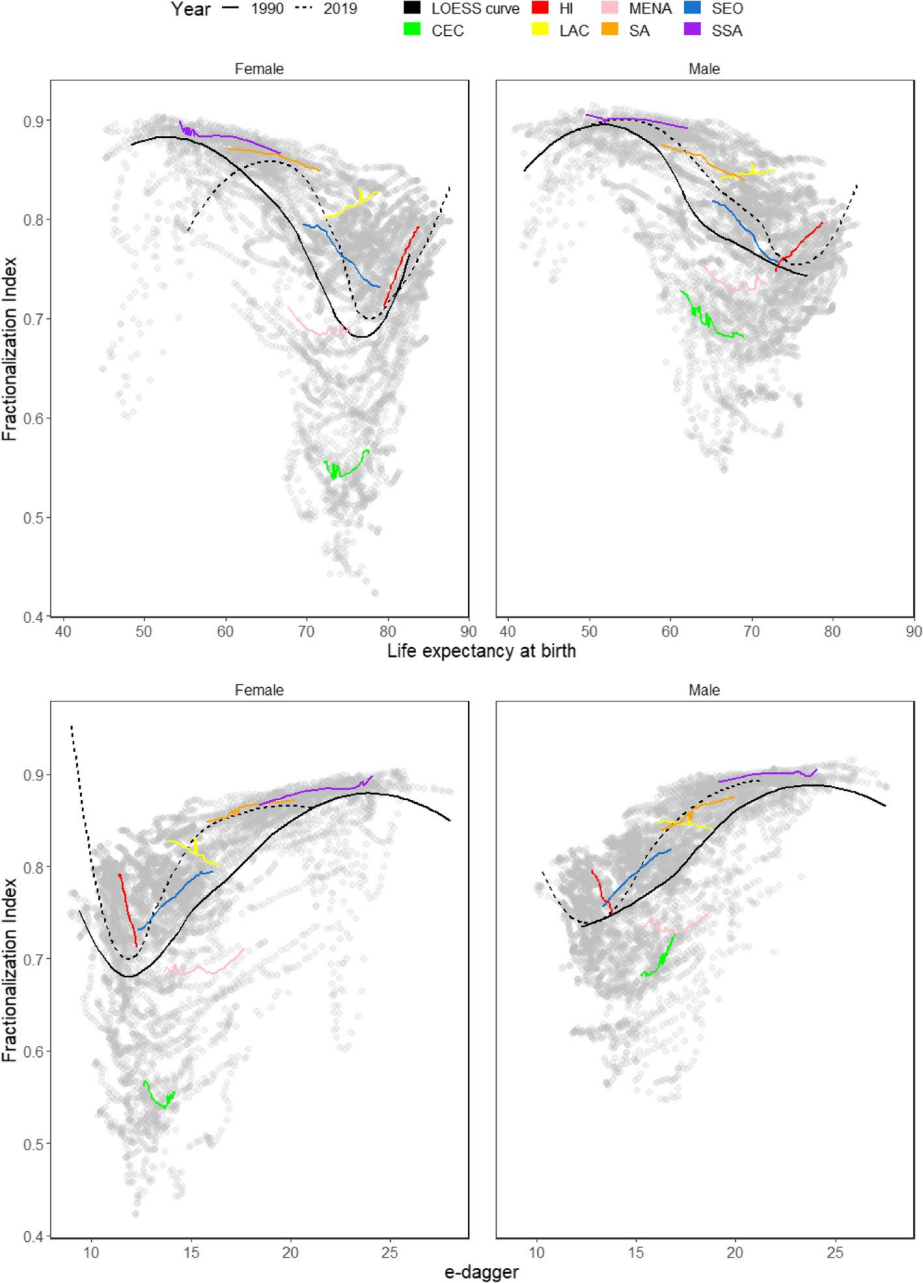
Relation between fractionalization index, life expectancy and e-dagger, by sex and region (1990 – 2019). Source: Global Burden of Disease (GBD/IHME). Note: Central Europe, Eastern Europe, and Central Asia (CEC); High-income (HI); Latin America and the Caribbean (LAC); North Africa and Middle East (MENA); South Asia (SA); Southeast Asia, East Asia, and Oceania (SEO); and Sub-Saharan Africa (SSA)

Across countries, the relationship between CoD diversity and life expectancy is non-monotonic, as shown by the population-weighted LOESS curve for these two indicators in 1990 and 2019 (upper panels of Fig. 3). At very low levels of life expectancy, the LOESS curve starts increasing. After peaking, it starts declining for a long range of life expectancy values. Lastly, the LOESS curve rebounds and increases again at even higher life expectancy values. This relationship holds both for women and men, but the turning points of the curves differ by sex and year. When moving from 1990 to 2019, the LOESS curves shift to the right, owing to the generalized increases in life expectancy during the last decades. The spread of the scatterplots is higher for specific ranges of life expectancy (around 65–80 for women and 60–75 for men). However, it is considerably smaller at the lower and upper extremes of the life expectancy distribution.

In Fig. 3, we also show the year-to-year joint trajectories in CoD diversity and life expectancy of the seven regions. Since the country-level observations are clustered according to the region to which they belong (i.e., the CoD diversity and life expectancy values of countries belonging to the same region tend to cluster together in the scatterplots shown in Fig. 3), these regional trajectories represent what has occurred in the corresponding average country reasonably well. The regional trajectories over time also show a non-monotonic relationship between CoD diversity and life expectancy. For some regions, the relationship is negative (SEO, SA), and for others, it is positive (LAC, HI). SSA, MENA, and CEC regions seem to lie on turning points, where the relationship shifts from positive to negative or vice versa.

Analogous patterns are found when exploring the relationship between CoD diversity and life disparity (bottom panels in Fig. 3). Again, we find non-monotonic LOESS curves, whose turning points differ by sex and year. In this case, the LOESS curves shift to the left-over time, owing to the generalized decreases in life disparity during the last decades. The spread of the scatterplot is much smaller at the extremes of the life disparity distribution than in the middle, where it is much higher.

## Discussion

This study documents the levels and trends in CoD diversity, measured with the fractionalization index ($F$), within regions and countries around the world between 1990 and 2019. CoD diversity has declined across world regions, except for LAC, HI, and women in CEC. Changes in mortality at adult and older ages have been mostly responsible for CoD diversity dynamics, except for the regions of SA and SSA, where infant and child mortality still play a non-negligible role. The relationship between CoD diversity, life expectancy, and life disparity is strongly non-monotonic, with turning points differing by sex and indicator.

Over the three decades analyzed, all regions of the world showed a sharp decline in mortality rates from cardiovascular disease, thus indicating that all regions of the world have already initiated, to some extent, the so-called ‘cardiovascular revolution’ [29]. Despite such declines, cardiovascular disease is still the leading cause of death worldwide [30], and its dynamics have been the main driving force for CoD diversity changes over time. In HI, LAC, and women in CEC, the decline in mortality rates from cardiovascular diseases is translated into a reduction of their share of total mortality, making room for the appearance of neoplasms or neurological disorders, thus increasing CoD diversity. These patterns cohere with the findings of Bergeron et al. [11] for a group of 15 low-mortality countries (Additional file 3.4) and extend them to a global scale. In the remaining world regions, the share of cardiovascular deaths among total deaths increases (despite the declines in cardiovascular mortality rates), thus depressing the corresponding CoD diversity levels.

The Epidemiological Transition Theory proposed by Omran [7] suggests that when mortality declines, the composition of CoD shifts from a majority of communicable deaths towards a majority of non-communicable deaths. During such transition, CoD diversity would begin at initially low levels (i.e., when most deaths were attributable to communicable diseases), then start increasing (as deaths shift from communicable diseases to non-communicable diseases), and finally decline to low levels again (when most deaths are non-communicable diseases). Such stylized trends roughly square with some of the patterns identified in Fig. 3 (e.g., SSA and SA seem to be somewhere in the transition from communicable to non-communicable mortality, with high CoD diversity levels), but not all. Rather than following an inverted U shape trajectory converging towards ‘uniformity’, where most deaths are attributable to a reduced number of causes, our findings suggest that, with further mortality declines, CoD diversity can bounce upwards once again, as observed especially for HI. Such trends might be partially attributable to the so-called ‘age of delayed degenerative diseases’ [31] as an extension of the original Epidemiological Transition Theory [7]. The case of LAC also stands out. The region presents a high diversity in the mortality profile by cause, especially as a result of an overlapping of stages in the process of epidemiological transition (with a high proportion of deaths from infectious diseases and external causes), which in turn is partly attributable to the large socio-economic inequality and poverty levels entrenched in the region. However, at the same time, it shows a reduction in mortality from cardiovascular diseases and increased CoD diversity over time. This result is corroborated by other mortality studies [32], thus highlighting the complexity of the epidemiological transition process in this world region.

The non-monotonic relationship between CoD diversity and life expectancy aligns with the Health Transition Theory suggested by Frenk et al. [32] and later adopted by Vallin and Meslé [29]. The introduction of new technologies (e.g., the introduction of pacemakers) or the occurrence of external shocks (e.g., the collapse of the Soviet Union) lead to successive waves of health divergence-convergence cycles, either across or within countries [33–35]. Likewise, the relationships reported in Fig. 3 also cohere with the patterns described by Mackenbach [36], in which disease incidence typically follows a rise-and-fall trajectory – with the ‘length’ and ‘height’ of such trajectories being disease-specific. The successive appearance and disappearance of diseases would naturally lead to the emergence of CoD diversity waves documented here.

When life expectancy increases from ‘low’ (e.g., around 50 years) to ‘medium–high’ values (e.g., around 70 years) – or, using Omran’s terminology, when countries transition from the ‘age of receding pandemics’ towards ‘the age of degenerative and man-made diseases’ – CoD diversity tends to decline because the share of deaths by communicable diseases shrinks considerably. However, the range of $F$ values in those countries is wide (somewhere between 0.45 and 0.85) and varies considerably across geographical regions and sex (Fig. 3). Such variability suggests that, as mortality declines, there is no universal pathway towards low CoD diversity, thus casting doubts on the ability of Epidemiological Transition Theory to predict prospective CoD dynamics among low- and middle-income countries – a finding that resonates with Sudharsanan et al. [37].

The findings reported in this paper suggest that, among longevity vanguard countries, further increases in life expectancy are associated with decreasing lifespan inequality but *increasing* CoD diversity. Despite the postponement and increasing predictability of the ages at which individuals die, contemporary aging populations are composed of an increasingly heterogenous mix of robust and frail individuals [6, 15], thus increasing the diversity of health profiles among older persons. This issue could potentially complicate further improvements in longevity. The existing health inequalities further reinforce these dynamics among social strata, which are pervasive and tend to increase over time [19].

In most cases, CoD diversity is higher among men than among women; that is, the mortality profile of men is more diverse than that of women. Furthermore, men also have a lower life expectancy and greater life disparity than women from the same region. Thus, men not only live shorter lives, but the ages in (and causes from) which they die are also more unpredictable than for women. These differences are mostly explained by the high prevalence of external causes in male mortality and the prevalence of other causes, especially chronic diseases, at relatively young ages. However, the gender gap in CoD diversity tends to decrease over time for most regions, with the exception of SEO and SSA.

This study has some limitations. Diversity indicators are sensitive to the number of categories one works with [12, 13, 38]. To reduce this problem, we consistently use the same CoD grouping across regions during the entire period. In addition, we tested the sensitivity of our results to alternative groupings, finding that, even if the magnitude changes marginally, the trends remain similar overall (Additional file 3.5). Another potential problem is the comparability of the death records over time. However, the GBD Project strives to guarantee coherence and consistency in the database, partially circumventing this issue. On the other hand, our results might be influenced by the assumptions that GBD makes in estimating mortality information, especially in countries with limited data sources [39]. An exhaustive analysis of the effects of such assumptions on CoD diversity trends is beyond the scope of this paper, but a large part of the problem is minimized when one works with large regions instead of countries. Notwithstanding these limitations, since our objective is to compare regions at a global level and over time, the GBD database is among the best data sources that are currently available. A fourth problem that could affect our results is that when death retreats to older ages, co-morbidity becomes more prevalent, thus increasing the difficulty in identifying the underlying cause of death [40–44]. Future research should go beyond the analysis of single causes of death and adopt the more comprehensive multiple causes of death approaches [43, 45–49]. Lastly, the covid-19 outbreak has not been captured in our analyses. The impact that the pandemic might have had on the CoD diversity indicators investigated here is potentially large, as the structure of causes of death might have shifted significantly in many countries around the globe. Future research should determine not only the size but also the direction of such changes.

## Conclusion

The analysis of CoD diversity dynamics is an important yet highly understudied area of research. The more homogeneous a mortality profile is, the more precise and efficient public health policies can be, thus facilitating more significant improvements and saving costs to health systems. Alternatively, greater diversification in mortality profiles implies lower predictability of individual eventual cause of death. With a clearer understanding of the similarity or lack of uniformity in the patterns of change over time and of the relationship between those patterns and the underlying causes of death in a population, we can improve our ability to predict and influence future patterns of mortality change.

The findings reported in this paper should contribute toward a better understanding of contemporary health dynamics against a backdrop of generalized population aging. The diversity of pathways to low-mortality highlights the limitations of classical Epidemiological Transition Theory and warns against using over-simplistic narratives based on deterministic stages – especially in the case of low- and middle-income countries, where several of such stages seem to be occurring at the same time. CoD profiles reflect the living conditions across and within countries, and they might be greatly affected by the emergence of pandemics (as evidenced by the covid-19 pandemic) as well as geopolitical tensions, economic instability, wars, and increasing inequalities, natural resources depletion, and environmental degradation. These factors (alone or in conjunction with each other) can cause reversals in death rates and contribute to a widening of existing health inequalities – so the future trends in CoD diversity are particularly uncertain.

## Supplementary Information

**Additional file 1.** Regional and country classification.

**Additional file 2.** Mathematical appendix.

**Additional file 3.** Results.

## Data Availability

The datasets analysed during the current study are available in https://vizhub.healthdata.org/gbd-results/. The scripts generated during the current study are available in the Git-hub repository (https://github.com/healinproject/CoD-Diversity). We conducted our analyses using the open-source statistical software R (version R-4.1.0).

## Abbreviations

COD: Causes of death
F: Fractionalization index
LOESS: Locally estimated scatterplot smoothing
CEC: Central Europe, Eastern Europe, and Central Asia
HI: High-income
LAC: Latin America and the Caribbean
MENA: North Africa and Middle East
SA: South Asia
SEO: Southeast Asia, East Asia, and Oceania
SSA: Sub-Saharan Africa

## Footnotes

1 This is also known as the Simpson’s diversity index [50], which, depending on the context one is dealing with, is defined as the likelihood that two random observations belong to different categories, or as the likelihood that they belong to the same category.
