# Supplementary material for: Levels, trends, and determinants of cause-of-death diversity in a global perspective: 1990–2019
Source: BMC Public Health. 2023 Apr 5;23:650. doi: 10.1186/s12889-023-15502-4 (PMC10072917; doi:10.1186/s12889-023-15502-4)
Supplement: Supplementary file 1 — Additional file 1. Regional and country classification. [file 12889_2023_15502_MOESM1_ESM.docx]

# **ADDITIONAL FILE 1. REGIONAL AND COUNTRY CLASSIFICATION**

We used the same regional and country classification as the Global Burden of Disease (GBD) Project 2019 [10, 31]. This comprises 204 countries and territories and seven super-regions: Central Europe, Eastern Europe, and Central Asia (CEC); High-income (HI); Latin America and the Caribbean (LAC); North Africa and Middle East (MENA); South Asia (SA); Southeast Asia, East Asia, and Oceania (SEO); and Sub-Saharan Africa (SSA). Estimates at the global/world level are also provided. The classification can be visualized at <https://www.iapb.org/learn/vision-atlas/about/definitions-and-regions/> (accessed on Oct 21, 2022) and is summarized in the tables below.

### Table A1 - Country classification, according Global Burden of Disease Study 2019

| **Central Europe, eastern Europe and central Asia (CEC)** | |
| --- | --- |
| **location_id** | **location_name** |
| 43 | Albania |
| 33 | Armenia |
| 34 | Azerbaijan |
| 57 | Belarus |
| 44 | Bosnia and Herzegovina |
| 45 | Bulgaria |
| 46 | Croatia |
| 47 | Czechia |
| 58 | Estonia |
| 35 | Georgia |
| 48 | Hungary |
| 36 | Kazakhstan |
| 37 | Kyrgyzstan |
| 59 | Latvia |
| 60 | Lithuania |
| 61 | Moldova |
| 38 | Mongolia |
| 50 | Montenegro |
| 49 | North Macedonia |
| 51 | Poland |
| 52 | Romania |
| 62 | Russia |
| 53 | Serbia |
| 54 | Slovakia |
| 55 | Slovenia |
| 39 | Tajikistan |
| 40 | Turkmenistan |
| 63 | Ukraine |
| 41 | Uzbekistan |
| **High-income countries (HI)** | |
| 74 | Andorra |
| 97 | Argentina |
| 71 | Australia |
| 75 | Austria |
| 76 | Belgium |
| 66 | Brunei |
| 101 | Canada |
| 98 | Chile |
| 77 | Cyprus |
| 78 | Denmark |
| 79 | Finland |
| 80 | France |
| 81 | Germany |
| 82 | Greece |
| 349 | Greenland |
| 83 | Iceland |
| 84 | Ireland |
| 85 | Israel |
| 86 | Italy |
| 67 | Japan |
| 87 | Luxembourg |
| 88 | Malta |
| 367 | Monaco |
| 89 | Netherlands |
| 72 | New Zealand |
| 90 | Norway |
| 91 | Portugal |
| 396 | San Marino |
| 69 | Singapore |
| 68 | South Korea |
| 92 | Spain |
| 93 | Sweden |
| 94 | Switzerland |
| 95 | United Kingdom |
| 102 | United States of America |
| 99 | Uruguay |
| **Latin America and Caribbean (LAC)** | |
| 105 | Antigua and Barbuda |
| 106 | Bahamas |
| 107 | Barbados |
| 108 | Belize |
| 305 | Bermuda |
| 121 | Bolivia |
| 135 | Brazil |
| 125 | Colombia |
| 126 | Costa Rica |
| 109 | Cuba |
| 110 | Dominica |
| 111 | Dominican Republic |
| 122 | Ecuador |
| 127 | El Salvador |
| 112 | Grenada |
| 128 | Guatemala |
| 113 | Guyana |
| 114 | Haiti |
| 129 | Honduras |
| 115 | Jamaica |
| 130 | Mexico |
| 131 | Nicaragua |
| 132 | Panama |
| 136 | Paraguay |
| 123 | Peru |
| 385 | Puerto Rico |
| 393 | Saint Kitts and Nevis |
| 116 | Saint Lucia |
| 117 | Saint Vincent and the Grenadines |
| 118 | Suriname |
| 119 | Trinidad and Tobago |
| 133 | Venezuela |
| 422 | Virgin Islands |
| **North Africa and Middle East (MENA)** | |
| 160 | Afghanistan |
| 139 | Algeria |
| 140 | Bahrain |
| 141 | Egypt |
| 142 | Iran |
| 143 | Iraq |
| 144 | Jordan |
| 145 | Kuwait |
| 146 | Lebanon |
| 147 | Libya |
| 148 | Morocco |
| 150 | Oman |
| 149 | Palestine |
| 151 | Qatar |
| 152 | Saudi Arabia |
| 522 | Sudan |
| 153 | Syria |
| 154 | Tunisia |
| 155 | Turkey |
| 156 | United Arab Emirates |
| 157 | Yemen |
| **South Asia (SA)** | |
| 161 | Bangladesh |
| 162 | Bhutan |
| 163 | India |
| 164 | Nepal |
| 165 | Pakistan |
| **Southeast Asia, east Asia and Oceania (SEO)** | |
| 298 | American Samoa |
| 10 | Cambodia |
| 6 | China |
| 320 | Cook Islands |
| 25 | Federated States of Micronesia |
| 22 | Fiji |
| 351 | Guam |
| 11 | Indonesia |
| 23 | Kiribati |
| 12 | Laos |
| 13 | Malaysia |
| 14 | Maldives |
| 24 | Marshall Islands |
| 183 | Mauritius |
| 15 | Myanmar |
| 369 | Nauru |
| 374 | Niue |
| 7 | North Korea |
| 376 | Northern Mariana Islands |
| 380 | Palau |
| 26 | Papua New Guinea |
| 16 | Philippines |
| 27 | Samoa |
| 186 | Seychelles |
| 28 | Solomon Islands |
| 17 | Sri Lanka |
| 8 | Taiwan (Province of China) |
| 18 | Thailand |
| 19 | Timor-Leste |
| 413 | Tokelau |
| 29 | Tonga |
| 416 | Tuvalu |
| 30 | Vanuatu |
| 20 | Vietnam |
| **Sub-Saharan Africa (SSA)** | |
| 168 | Angola |
| 200 | Benin |
| 193 | Botswana |
| 201 | Burkina Faso |
| 175 | Burundi |
| 203 | Cabo Verde |
| 202 | Cameroon |
| 169 | Central African Republic |
| 204 | Chad |
| 176 | Comoros |
| 170 | Congo |
| 205 | Cote d'Ivoire |
| 171 | Democratic Republic of the Congo |
| 177 | Djibouti |
| 172 | Equatorial Guinea |
| 178 | Eritrea |
| 197 | Eswatini |
| 179 | Ethiopia |
| 173 | Gabon |
| 206 | Gambia |
| 207 | Ghana |
| 208 | Guinea |
| 209 | Guinea-Bissau |
| 180 | Kenya |
| 194 | Lesotho |
| 210 | Liberia |
| 181 | Madagascar |
| 182 | Malawi |
| 211 | Mali |
| 212 | Mauritania |
| 184 | Mozambique |
| 195 | Namibia |
| 213 | Niger |
| 214 | Nigeria |
| 185 | Rwanda |
| 215 | Sao Tome and Principe |
| 216 | Senegal |
| 217 | Sierra Leone |
| 187 | Somalia |
| 196 | South Africa |
| 435 | South Sudan |
| 189 | Tanzania |
| 218 | Togo |
| 190 | Uganda |
| 191 | Zambia |
| 198 | Zimbabwe |
