# Supplementary material for: Levels, trends, and determinants of cause-of-death diversity in a global perspective: 1990–2019
Source: BMC Public Health. 2023 Apr 5;23:650. doi: 10.1186/s12889-023-15502-4 (PMC10072917; doi:10.1186/s12889-023-15502-4)
Supplement: Supplementary file 2 — Additional file 2. Mathematical appendix. [file 12889_2023_15502_MOESM2_ESM.docx]

# **ADDITIONAL FILE 2. MATHEMATICAL APPENDIX**

## **Fractionalization index**

The fractionalization index ($F$) can be expressed as:

$$F=1- \sum_{i=1}^{k} p_{i}^{2} [1]$$

where $p_{i}$ is the share of deaths from cause $i$ taken from the life table, and $k$ is the number of causes of death. By construction, the sum of the different $p_{i}$ equals 1. Assuming all deaths are classified in a list of mutually exclusive causes, the index is defined as the probability that two randomly chosen deaths are attributable to different causes. The Fractionalization index is also known as the Simpson index of diversity, which has been widely used in ecology studies to assess the extent of biodiversity in an ecosystem.

## **Life expectancy at birth**

Life expectancy at birth can be defined as the average number of years a cohort would expect to live if it experienced the prevailing mortality rates, from birth to death, in a period of time $t$. Formally, it is calculated as:

$$e_{0}\left( t \right)= \int_{0}^{\omega} l\left( a, t \right)da [2]$$

where $l\left( a, t \right)$ is the probability of survival from birth to age $a$ at time $t$ from the life table, and $\omega$ is the maximum lifespan. More generally, the life expectancy at age $x$ in time $t$ is defined as:

$$e_{x}\left( t \right)= \int_{x}^{\omega} l\left( a, t \right)da [3]$$

## **Life disparity**

The dispersion of age at death is measured by the life disparity indicator, e-dagger ($e_{0}^{\dagger}$), which in turn can be defined as the average remaining life expectancy weighted by the age distribution of deaths:

$$e_{0}^{\dagger}\left( t \right)= \int_{0}^{\omega} e_{x}\left( t \right)f\left( x,t \right)dx [4]$$

where $e_{x}\left( t \right)$ is the remaining life expectancy at age $x$ and time $t$ (see equation [3]), and $f\left( x,t \right)$ is the life table distribution of deaths at age $x$ and time $t$.

## **Decomposition**

The decomposition method used here is based on the line integral model (Horiuchi et al. 2008). Suppose that the Fractionalization index *F* is a differentiable function of n covariates (age-cause specific mortality rate) denoted by the vector A= [$x_{1}, x_{2}, \ldots, x_{n}$]^T^. Assume that *F* and A depend on the underlying dimension *t*, which is time in this case, and that we have observations available in two time points, 1990 and 2019. Assuming that A is a differentiable function of *t* between 1990 and 2019, the difference in *F* over time can be expressed as a sum of the effects of the covariates:

$\Delta F= F_{2019}- F_{1990}= \sum_{i=1}^{n} c_{i}$ where $c_{i}= \int_{x_{i1}}^{x_{i2}} \frac{\partial F}{\partial x_{i1}}{dx}_{i}$ [5]

The decomposition was calculated from the R package "DemoDecomp", authored by Tim Riffe, with documentation available at:

<https://cran.r-project.org/web/packages/DemoDecomp/DemoDecomp.pdf>.

## **Uncertainty analysis**

The GBD provides its mortality estimates with the 95% uncertainty interval. Thus, the proportion of deaths by cause in the life table used to calculate the Fractionalization indicator will consider both the uncertainty in estimating the *dx* function and the uncertainty in estimating the proportions of deaths by causes before correction by age structure based on the life table.

We assumed that the proportion of deaths by cause from the life table follows a log-normal distribution, since it cannot assume values less than zero^[[1]](#footnote-1)^. Thus, we approximated the standard deviation by dividing the 95% uncertainty interval range by 3.92. We randomly drew 20,000 samples, using Monte Carlo simulation techniques, for the proportion of each of the 21 cause groups and obtained the uncertainty intervals from the selected percentiles.

We used the uncertainty intervals for each of the causes to obtain the upper and lower bounds of the fractionalization index. The fractionalization index is the probability that two randomly chosen deaths are attributable to different causes and, therefore, cannot also assume values less than zero. Thus, to calculate the uncertainty around the Fractionalization index, we assume that it follows a log-normal distribution, with mean μ and standard deviation s:

$\mu=\log\left( \frac{m^{2}}{\sqrt{s^{2}+m^{2}}} \right) \mathrm{and} \sigma^{2}=\log\left( 1+\frac{s^{2}}{m^{2}} \right)$ [6]

Since $m$ is the mean and $s$ is the standard deviation of the normal distribution, we approximated the standard deviation by dividing the range of the 95% uncertainty interval by 3.92. We randomly drew 20,000 samples from these values, using Monte Carlo simulation techniques to obtain the uncertainty intervals for the index from the selected percentiles.

Finally, we also incorporated the uncertainty in calculating the contribution of each of the causes on the variation of the fractionalization index in the Horiuchi decomposition. Since the contributions of each cause can take both negative and positive values on the variation of the fractionalization index, we assume that the contributions follow a normal distribution. We randomly drew 20,000 samples, using Monte Carlo simulation techniques, for the proportion of each of the 21 cause groups and obtained the uncertainty intervals from the selected percentiles.

1. See Liu L, Villavicencio F, Yeung D, Perin J, Lopez G, Strong KL, et al. National, regional, and global causes of mortality in 5-19-year-olds from 2000 to 2019: a systematic analysis. Lancet Glob Health [Internet]. 2022;10(3): e337–47. Available from: <http://dx.doi.org/10.1016/S2214-109X(21)00566-0> [↑](#footnote-ref-1)
