# Supplementary material for: Levels, trends, and determinants of cause-of-death diversity in a global perspective: 1990–2019
Source: BMC Public Health. 2023 Apr 5;23:650. doi: 10.1186/s12889-023-15502-4 (PMC10072917; doi:10.1186/s12889-023-15502-4)
Supplement: Supplementary file 3 — Additional file 3. Results. [file 12889_2023_15502_MOESM3_ESM.docx]

# **ADDITIONAL FILE 3. RESULTS**

## **Deaths distribution by cause from life table**

Tables A2 to A5 present the distribution of deaths by cause of life table used in the construction of Figure 1 from the main manuscript with their respective uncertainty intervals calculated as described in Additional File Section 2.5.

### Table A2 - Deaths distribution by cause from life table with 95% uncertainty interval – Females, 1990

|  | **SEO** | **CEC** | **HI** | **LAC** | **MENA** | **SA** | **SSA** |
| --- | --- | --- | --- | --- | --- | --- | --- |
| **Non-communicable diseases** | | | | | | | |
| Cardiovascular diseases | 37.43  (33.99 to 41.10) | 65.11  (62.35 to 67.91) | 48.08  (45.28 to 51.01) | 39.28  (36.75 to 41.89) | 51.36  (46.17 to 57.03) | 22.18  (19.22 to 25.56) | 22.49  (18.64 to 27.01) |
| Neoplasms | 13.11  (12.38 to 13.87) | 14.13  (13.30 to 14.98) | 20.85  (18.93 to 22.92) | 14.32  (13.22 to 15.48) | 8.25  (7.83 to 8.67) | 6.23  (5.38 to 7.18) | 7.10  (6.07 to 8.26) |
| Diabetes and kidney diseases | 3.63  (3.45 to 3.82) | 1.69  (1.62 to 1.77) | 4.21  (3.93 to 4.51) | 8.62  (8.03 to 9.24) | 7.06  (7.00 to 7.11) | 3.01  (2.65 to 3.40) | 4.41  (3.66 to 5.26) |
| Chronic respiratory diseases | 19.00  (18.07 to 19.97) | 3.28  (2.78 to 3.85) | 3.82  (3.70 to 3.94) | 5.35  (5.30 to 5.40) | 4.89  (4.59 to 5.21) | 11.43  (10.38 to 12.57) | 3.70  (3.62 to 3.78) |
| Mental disorders | 0.00  (0.00 to 0.00) | 0.00  (0.00 to 0.00) | 0.00  (0.00 to 0.00) | 0.00  (0.00 to 0.00) | 0.00  (0.00 to 0.00) | 0.00  (0.00 to 0.00) | 0.00  (0.00 to 0.00) |
| Neurological disorders | 3.43  (1.58 to 6.53) | 3.69  (1.42 to 7.91) | 7.03  (3.04 to 14.18) | 5.15  (2.14 to 10.42) | 3.68  (1.66 to 7.03) | 1.57  (1.00 to 2.33) | 2.13  (1.20 to 3.53) |
| Substance use disorders | 0.17  (0.16 to 0.17) | 0.46  (0.44 to 0.47) | 0.14  (0.13 to 0.16) | 0.10  (0.09 to 0.11) | 0.06  (0.06 to 0.06) | 0.05  (0.04 to 0.05) | 0.05  (0.04 to 0.06) |
| Digestive diseases | 3.91  (3.70 to 4.13) | 2.77  (2.64 to 2.90) | 4.13  (3.84 to 4.44) | 5.23  (4.90 to 5.58) | 5.95  (5.84 to 6.06) | 4.12  (3.73 to 4.55) | 4.23  (3.72 to 4.77) |
| Musculoskeletal disorders | 0.11  (0.09 to 0.14) | 0.10  (0.07 to 0.15) | 0.41  (0.30 to 0.55) | 0.30  (0.21 to 0.41) | 0.09  (0.07 to 0.11) | 0.20  (0.20 to 0.21) | 0.08  (0.07 to 0.09) |
| Skin and subcutaneous diseases | 0.15  (0.15 to 0.15) | 0.05  (0.03 to 0.09) | 0.22  (0.14 to 0.32) | 0.32  (0.23 to 0.44) | 0.06  (0.04 to 0.10) | 0.14  (0.12 to 0.16) | 0.17  (0.16 to 0.19) |
| Other non-communicable diseases | 1.15  (0.98 to 1.34) | 1.00  (0.95 to 1.05) | 1.38  (1.35 to 1.42) | 1.65  (1.51 to 1.81) | 1.71  (1.30 to 2.21) | 1.28  (1.28 to 1.29) | 1.47  (1.13 to 1.88) |
| **Communicable, maternal, neonatal, and nutritional diseases** | | | | | | | |
| Neglected tropical diseases and malaria | 0.18  (0.10 to 0.30) | 0.02  (0.01 to 0.05) | 0.03  (0.03 to 0.04) | 0.63  (0.42 to 0.90) | 0.34  (0.17 to 0.64) | 1.32  (0.73 to 2.18) | 6.13  (4.64 to 7.98) |
| HIV/AIDS and sexually transmitted infections | 0.05  (0.03 to 0.09) | 0.06  (0.06 to 0.06) | 0.09  (0.08 to 0.10) | 0.24  (0.20 to 0.28) | 0.04  (0.02 to 0.08) | 0.13  (0.09 to 0.20) | 4.30  (3.56 to 5.13) |
| Respiratory infections and tuberculosis | 7.40  (7.19 to 7.63) | 2.32  (2.27 to 2.36) | 4.73  (4.52 to 4.94) | 7.70  (7.44 to 7.96) | 5.09  (4.71 to 5.48) | 11.88  (10.85 to 12.96) | 16.01  (14.41 to 17.71) |
| Enteric infections | 1.90  (1.10 to 3.06) | 0.25  (0.23 to 0.27) | 0.12  (0.11 to 0.12) | 2.55  (2.31 to 2.82) | 1.52  (0.94 to 2.31) | 19.75  (14.81 to 25.99) | 11.82  (7.95 to 16.87) |
| Other infectious diseases | 1.26  (1.06 to 1.49) | 0.26  (0.24 to 0.28) | 0.20  (0.19 to 0.21) | 0.53  (0.50 to 0.57) | 1.95  (1.37 to 2.68) | 3.85  (3.78 to 3.92) | 4.73  (4.10 to 5.42) |
| Maternal and neonatal disorders | 1.62  (1.57 to 1.68) | 0.88  (0.87 to 0.89) | 0.41  (0.37 to 0.45) | 2.02  (1.96 to 2.08) | 2.94  (2.90 to 2.98) | 4.78  (4.07 to 5.59) | 4.58  (4.14 to 5.07) |
| Nutritional deficiencies | 0.64  (0.60 to 0.68) | 0.04  (0.04 to 0.04) | 0.18  (0.18 to 0.18) | 1.86  (1.81 to 1.92) | 0.33  (0.21 to 0.49) | 2.45  (1.53 to 3.73) | 2.09  (2.01 to 2.17) |
| **Injuries** | | | | | | | |
| Transport injuries | 1.04  (1.03 to 1.05) | 0.83  (0.80 to 0.86) | 0.88  (0.79 to 0.97) | 1.18  (1.11 to 1.26) | 1.81  (1.71 to 1.90) | 0.68  (0.60 to 0.77) | 1.25  (1.19 to 1.31) |
| Self-harm and interpersonal violence | 1.69  (1.68 to 1.70) | 1.22  (1.16 to 1.29) | 0.88  (0.79 to 0.99) | 0.76  (0.71 to 0.82) | 0.52  (0.52 to 0.53) | 1.23  (1.09 to 1.38) | 1.31  (1.15 to 1.49) |
| Unintentional injuries | 2.12  (2.07 to 2.17) | 1.85  (1.80 to 1.91) | 2.20  (2.11 to 2.30) | 2.19  (2.10 to 2.27) | 2.36  (2.30 to 2.42) | 3.71  (3.49 to 3.95) | 1.95  (1.76 to 2.16) |

Source: Global Burden of Disease (GBD/IHME). Note: 95% uncertainty interval in parenthesis. Central Europe, Eastern Europe, and Central Asia (CEC); High-income (HI); Latin America and the Caribbean (LAC); North Africa and Middle East (MENA); South Asia (SA); Southeast Asia, East Asia, and Oceania (SEO); and Sub-Saharan Africa (SSA).

### Table A3 - Deaths distribution by cause from life table with 95% uncertainty interval – Females, 2019

|  | **SEO** | **CEC** | | **HI** | | **LAC** | | **MENA** | | **SA** | | **SSA** | |  |
| --- | --- | --- | --- | --- | --- | --- | --- | --- | --- | --- | --- | --- | --- | --- |
| **Non-communicable diseases** | | | | | | | | | | | | | | |
| Cardiovascular diseases | 47.11  (42.53 to 52.02) | | 63.84  (59.65 to 68.32) | | 35.80  (32.88 to 38.87) | | 33.53  (30.72 to 36.55) | | 53.35  (48.68 to 58.33) | | 30.63  (26.95 to 34.76) | | 29.12  (25.10 to 33.58) | |
| Neoplasms | 15.52  (14.78 to 16.28) | | 15.13  (14.62 to 15.66) | | 23.36  (20.42 to 26.58) | | 15.93  (14.58 to 17.38) | | 11.03  (10.52 to 11.57) | | 9.78  (8.85 to 10.79) | | 10.22  (9.13 to 11.42) | |
| Diabetes and kidney diseases | 5.38  (5.11 to 5.66) | | 2.64  (2.58 to 2.71) | | 5.74  (5.23 to 6.30) | | 12.04  (11.08 to 13.07) | | 9.01  (8.73 to 9.31) | | 5.88  (5.17 to 6.66) | | 6.74  (5.83 to 7.78) | |
| Chronic respiratory diseases | 10.35  (10.13 to 10.58) | | 2.03  (1.77 to 2.32) | | 5.48  (5.47 to 5.49) | | 6.29  (6.08 to 6.50) | | 4.55  (4.37 to 4.74) | | 14.59  (13.89 to 15.31) | | 3.94  (3.87 to 4.01) | |
| Mental disorders | 0.00  (0.00 to 0.00) | | 0.00  (0.00 to 0.00) | | 0.00  (0.00 to 0.00) | | 0.00  (0.00 to 0.00) | | 0.00  (0.00 to 0.00) | | 0.00  (0.00 to 0.00) | | 0.00  (0.00 to 0.00) | |
| Neurological disorders | 6.85  (3.14 to 13.08) | | 5.55  (2.36 to 11.24) | | 11.81  (5.81 to 21.31) | | 8.54  (4.02 to 15.91) | | 5.64  (2.61 to 10.65) | | 3.87  (2.23 to 6.24) | | 3.85  (2.07 to 6.55) | |
| Substance use disorders | 0.05  (0.05 to 0.05) | | 0.42  (0.39 to 0.44) | | 0.48  (0.41 to 0.57) | | 0.09  (0.09 to 0.09) | | 0.08  (0.07 to 0.08) | | 0.06  (0.06 to 0.06) | | 0.07  (0.06 to 0.07) | |
| Digestive diseases | 2.94  (2.79 to 3.09) | | 3.79  (3.68 to 3.90) | | 4.45  (4.02 to 4.91) | | 5.45  (5.02 to 5.89) | | 4.92  (4.73 to 5.11) | | 3.96  (3.67 to 4.27) | | 4.74  (4.26 to 5.25) | |
| Musculoskeletal disorders | 0.22  (0.19 to 0.25) | | 0.13  (0.10 to 0.18) | | 0.48  (0.38 to 0.59) | | 0.40  (0.31 to 0.52) | | 0.15  (0.12 to 0.19) | | 0.45  (0.43 to 0.47) | | 0.13  (0.11 to 0.15) | |
| Skin and subcutaneous diseases | 0.20  (0.20 to 0.20) | | 0.10  (0.06 to 0.14) | | 0.36  (0.27 to 0.48) | | 0.61  (0.47 to 0.77) | | 0.09  (0.08 to 0.10) | | 0.18  (0.16 to 0.20) | | 0.24  (0.23 to 0.26) | |
| Other non-communicable diseases | 0.89  (0.87 to 0.91) | | 0.86  (0.81 to 0.92) | | 2.12  (2.00 to 2.25) | | 2.78  (2.69 to 2.87) | | 1.23  (1.15 to 1.32) | | 1.37  (1.36 to 1.37) | | 1.42  (1.21 to 1.67) | |
| **Communicable, maternal, neonatal, and nutritional diseases** | | | | | | | | | | | | | | |
| Neglected tropical diseases and malaria | 0.07  (0.05 to 0.11) | | 0.01  (0.00 to 0.01) | | 0.01  (0.00 to 0.02) | | 0.31  (0.18 to 0.48) | | 0.13  (0.05 to 0.29) | | 0.38  (0.23 to 0.59) | | 4.55  (2.76 to 7.03) | |
| HIV/AIDS and sexually transmitted infections | 0.22  (0.15 to 0.29) | | 0.28  (0.25 to 0.31) | | 0.06  (0.05 to 0.07) | | 0.42  (0.38 to 0.46) | | 0.15  (0.07 to 0.27) | | 0.27  (0.19 to 0.38) | | 6.24  (5.78 to 6.73) | |
| Respiratory infections and tuberculosis | 4.19  (3.99 to 4.41) | | 1.85  (1.82 to 1.87) | | 4.97  (4.65 to 5.29) | | 7.16  (6.72 to7.61) | | 3.59  (3.51 to 3.66) | | 7.45  (6.99 to 7.93) | | 12.87  (11.76 to 14.04) | |
| Enteric infections | 0.74  (0.40 to 1.27) | | 0.07  (0.06 to 0.08) | | 0.43  (0.41 to 0.46) | | 0.78  (0.74 to 0.84) | | 0.46  (0.26 to 0.78) | | 10.72  (6.57 to 16.58) | | 5.82  (3.52 to 9.05) | |
| Other infectious diseases | 0.30  (0.28 to 0.33) | | 0.14  (0.12 to 0.16) | | 0.17  (0.17 to 0.17) | | 0.22  (0.21 to 0.24) | | 0.51  (0.43 to 0.60) | | 1.29  (1.26 to 1.31) | | 2.00  (1.89 to 2.11) | |
| Maternal and neonatal disorders | 0.57  (0.56 to 0.58) | | 0.38  (0.36 to 0.39) | | 0.22  (0.18 to 0.25) | | 0.85  (0.84 to 0.85) | | 1.05  (1.00 to 1.09) | | 2.53  (2.34 to 2.73) | | 2.96  (2.93 to 2.99) | |
| Nutritional deficiencies | 0.48  (0.45 to 0.52) | | 0.03  (0.03 to 0.03) | | 0.27  (0.26 to 0.28) | | 0.91  (0.86 to 0.97) | | 0.19  (0.16 to 0.22) | | 0.41  (0.37 to 0.46) | | 1.12  (1.08 to 1.16) | |
| **Injuries** | | | | | | | | | | | | | | |
| Transport injuries | 0.84  (0.84 to 0.85) | | 0.51  (0.48 to 0.54) | | 0.48  (0.41 to 0.56) | | 0.78  (0.75 to 0.82) | | 1.28  (1.16 to 1.40) | | 0.78  (0.75 to 0.81) | | 1.09  (1.04 to 1.14) | |
| Self-harm and interpersonal violence | 0.65  (0.64 to 0.65) | | 0.87  (0.86 to 0.88) | | 0.68  (0.57 to 0.81) | | 0.64  (0.61 to 0.68) | | 1.17  (1.16 to 1.17) | | 0.89  (0.89 to 0.90) | | 0.76  (0.73 to 0.79) | |
| Unintentional injuries | 2.42  (2.23 to 2.63) | | 1.37  (1.34 to 1.39) | | 2.63  (2.42 to 2.85) | | 2.27  (2.11 to 2.42) | | 1.43  (1.28 to 1.59) | | 4.52  (4.37 to 4.67) | | 2.12  (1.98 to 2.28) | |

Source: Global Burden of Disease (GBD/IHME). Note: 95% uncertainty interval in parenthesis. Central Europe, Eastern Europe, and Central Asia (CEC); High-income (HI); Latin America and the Caribbean (LAC); North Africa and Middle East (MENA); South Asia (SA); Southeast Asia, East Asia, and Oceania (SEO); and Sub-Saharan Africa (SSA).

### Table A4 - Deaths distribution by cause from life table with 95% uncertainty interval – Males, 1990

|  | **SEO** | **CEC** | | **HI** | | **LAC** | | **MENA** | | **SA** | | **SSA** | |  |  |
| --- | --- | --- | --- | --- | --- | --- | --- | --- | --- | --- | --- | --- | --- | --- | --- |
| **Non-communicable diseases** | | | | | | | | | | | | | | | |
| Cardiovascular diseases | 32.60  (30.06 to 35.30) | | 50.13  (48.90 to 51.41) | | 40.95  (39.74 to 42.21) | | 33.85  (32.17 to 35.65) | | 46.34  (41.74 to 51.21) | | 24.03  (21.24 to 27.10) | | 17.16  (14.39 to 20.32) | |  |
| Neoplasms | 17.37  (16.74 to 18.03) | | 19.04  (18.70 to 19.39) | | 26.91  (25.97 to 27.86) | | 14.34  (13.88 to 14.81) | | 10.97  (10.57 to 11.37) | | 6.74  (6.16 to 7.37) | | 6.75  (5.95 to 7.64) | |  |
| Diabetes and kidney diseases | 2.78  (2.70 to 2.87) | | 1.33  (1.31 to 1.34) | | 3.27  (3.19 to 3.36) | | 5.83  (5.54 to 6.13) | | 5.11  (5.10 to 5.13) | | 3.32  (3.09 to 3.57) | | 4.23  (3.61 to 4.94) | |  |
| Chronic respiratory diseases | 17.79  (17.57 to 18.02) | | 5.51  (5.48 to 5.54) | | 6.08  (5.93 to 6.24) | | 5.94  (5.72 to 6.15) | | 5.62  (5.51 to 5.73) | | 13.51  (12.58 to 14.48) | | 4.03  (3.65 to 4.44) | |  |
| Mental disorders | 0.00  (0.00 to 0.00) | | 0.00  (0.00 to 0.00) | | 0.00  (0.00 to 0.00) | | 0.00  (0.00 to 0.00) | | 0.00  (0.00 to 0.00) | | 0.00  (0.00 to 0.00) | | 0.00  (0.00 to 0.00) | |  |
| Neurological disorders | 1.91  (1.03 to 3.24) | | 1.87  (0.85 to 3.63) | | 3.60  (1.71 to 6.85) | | 3.62  (1.59 to 7.11) | | 2.77  (1.39 to 5.01) | | 1.40  (0.91 to 2.06) | | 1.30  (0.84 to 1.93) | |  |
| Substance use disorders | 0.28  (0.25 to 0.31) | | 1.47  (1.46 to 1.47) | | 0.48  (0.47 to 0.49) | | 0.81  (0.79 to 0.84) | | 0.12  (0.12 to 0.13) | | 0.28  (0.26 to 0.30) | | 0.19  (0.18 to 0.19) | |  |
| Digestive diseases | 4.75  (4.71 to 4.80) | | 3.61  (3.54 to 3.67) | | 4.49  (4.34 to 4.65) | | 6.63  (6.30 to 6.97) | | 5.49  (5.48 to 5.49) | | 5.41  (5.08 to 5.76) | | 6.18  (5.83 to 6.54) | |  |
| Musculoskeletal disorders | 0.06  (0.06 to 0.06) | | 0.04  (0.03 to 0.05) | | 0.15  (0.12 to 0.19) | | 0.11  (0.09 to 0.13) | | 0.04  (0.04 to 0.05) | | 0.14  (0.13 to 0.14) | | 0.05  (0.05 to 0.05) | |  |
| Skin and subcutaneous diseases | 0.11  (0.09 to 0.13) | | 0.04  (0.02 to 0.08) | | 0.12  (0.05 to 0.23) | | 0.19  (0.10 to 0.34) | | 0.06  (1.27 to 2.24) | | 0.16  (0.12 to 0.23) | | 0.10  (0.09 to 0.10) | |  |
| Other non-communicable diseases | 1.22  (0.94 to 1.57) | | 1.09  (0.92 to 1.29) | | 1.21  (1.10 to 1.33) | | 1.63  (1.37 to 1.93) | | 1.71  (0.04 to 0.09) | | 1.24  (1.12 to 1.37) | | 1.74  (1.22 to 2.40) | |  |
| **Communicable, maternal, neonatal, and nutritional diseases** | | | | | | | | | | | | | | | |
| Neglected tropical diseases and malaria | 0.17  (0.09 to 0.31) | | 0.02  (0.01 to 0.06) | | 0.03  (0.02 to 0.05) | | 0.74  (0.48 to 1.09) | | 0.51  (0.24 to 0.97) | | 1.22  (0.65 to 2.10) | | 6.42  (4.62 to 8.68 | |  |
| HIV/AIDS and sexually transmitted infections | 0.06  (0.03 to 0.11) | | 0.16  (0.16 to 0.16) | | 0.53  (0.51 to 0.56) | | 0.54  (0.51 to 0.57) | | 0.04  (0.02 to 0.09) | | 0.10  (0.04 to 0.18) | | 3.37  (2.56 to 4.38) | |  |
| Respiratory infections and tuberculosis | 8.15  (8.07 to 8.23) | | 3.31  (3.27 to 3.34) | | 4.65  (4.59 to 4.72) | | 7.66  (7.51 to 7.82) | | 5.06  (4.85 to 5.28) | | 14.62  (13.62 to 15.63) | | 19.86  (17.79 to 22.12) | |  |
| Enteric infections | 2.02  (1.37 to 2.88) | | 0.28  (0.25 to 0.32) | | 0.08  (0.07 to 0.08) | | 2.34  (2.15 to 2.53) | | 1.40  (0.97 to 1.95) | | 11.77  (8.29 to 16.18) | | 10.24  (7.56 to 13.56) | |  |
| Other infectious diseases | 1.39  (1.10 to 1.74) | | 0.33  (0.30 to 0.35) | | 0.21  (0.18 to 0.24) | | 0.61  (0.56 to 0.66) | | 2.25  (1.49 to 3.28) | | 3.40  (3.10 to 3.72) | | 4.58  (3.66 to 5.68) | |  |
| Maternal and neonatal disorders | 1.51  (1.48 to 1.53) | | 1.09  (1.05 to 1.13) | | 0.49  (0.48 to 0.49) | | 2.07  (2.06 to 2.08) | | 2.71  (2.71 to 2.71) | | 3.64  (3.34 to 3.97) | | 3.17  (2.83 to 3.54) | |  |
| Nutritional deficiencies | 0.58  (0.54 to 0.63) | | 0.04  (0.03 to 0.04) | | 0.11  (0.11 to 0.11) | | 1.57  (1.57 to 1.58) | | 0.27  (0.18 to 0.39) | | 1.62  (1.25 to 2.05) | | 2.13  (2.11 to 2.14) | |  |
| **Injuries** | | | | | | | | | | | | | | | |
| Transport injuries | 2.44  (2.00 to 2.94) | | 2.59  (2.59 to 2.60) | | 2.08  (2.00 to 2.16) | | 3.53  (3.37 to 3.70) | | 4.44  (3.97 to 4.96) | | 1.72  (1.68 to 1.76) | | 2.40  (2.39 to 2.42) | |  |
| Self-harm and interpersonal violence | 1.85  (1.55 to 2.18) | | 3.77  (3.69 to 3.86) | | 2.24  (2.17 to 2.31) | | 4.39  (4.11 to 4.68) | | 1.27  (1.22 to 1.32) | | 1.77  (1.72 to 1.83) | | 3.43  (3.19 to 3.68) | |  |
| Unintentional injuries | 2.94  (2.81 to 3.07) | | 4.29  (4.25 to 4.33) | | 2.31  (2.26 to 2.35) | | 3.60  (3.46 to 3.74) | | 3.83  (3.74 to 3.93) | | 3.92  (3.84 to 4.01) | | 2.68  (2.64 to 2.72) | |  |

Source: Global Burden of Disease (GBD/IHME). Note: 95% uncertainty interval in parenthesis. Central Europe, Eastern Europe, and Central Asia (CEC); High-income (HI); Latin America and the Caribbean (LAC); North Africa and Middle East (MENA); South Asia (SA); Southeast Asia, East Asia, and Oceania (SEO); and Sub-Saharan Africa (SSA).

### Table A5 - Deaths distribution by cause from life table with 95% uncertainty interval – Males, 2019

|  | **SEO** | **CEC** | **HI** | **LAC** | **MENA** | **SA** | **SSA** |
| --- | --- | --- | --- | --- | --- | --- | --- |
| **Non-communicable diseases** | | | | | | | |
| Cardiovascular diseases | 41.78  (39.03 to 44.65) | 52.18  (49.56 to 54.89) | 31.16  (29.48 to 32.91) | 30.09  (27.99 to 32.26) | 48.36  (44.50 to 52.52) | 32.74  (29.63 to 36.11) | 22.12  (19.36 to 25.13) |
| Neoplasms | 21.98  (21.82 to 22.15) | 19.39  (19.03 to 19.74) | 29.68  (27.78 to 31.77) | 17.55  (16.69 to 18.45) | 14.29  (13.51 to 15.10) | 9.91  (9.43 to 10.42) | 10.32  (9.44 to 11.25) |
| Diabetes and kidney diseases | 4.28  (4.24 to 4.31) | 2.13  (2.12 to 2.13) | 5.36  (5.08 to 5.65) | 10.49  (9.93 to 11.07) | 6.87  (6.70 to 7.05) | 6.06  (5.64 to 6.51) | 6.54  (5.68 to 7.47) |
| Chronic respiratory diseases | 11.28  (10.71 to 11.87) | 3.33  (3.25 to 3.42) | 6.83  (6.49 to 7.18) | 6.46  (6.06 to 6.88) | 5.65  (5.53 to 5.77) | 15.15  (14.32 to 16.01) | 4.43  (4.00 to 4.89) |
| Mental disorders | 0.00  (0.00 to 0.00) | 0.00  (0.00 to 0.00) | 0.00  (0.00 to 0.00) | 0.00  (0.00 to 0.00) | 0.00  (0.00 to 0.00) | 0.00  (0.00 to 0.00) | 0.00  (0.00 to 0.00) |
| Neurological disorders | 3.30  (1.65 to 6.00) | 3.06  (1.51 to 5.61) | 7.32  (3.66 to 13.20) | 6.07  (3.03 to 10.90) | 4.58  (2.35 to 8.03) | 3.14  (1.84 to 5.04) | 2.46  (1.46 to 3.90) |
| Substance use disorders | 0.24  (0.21 to 0.28) | 1.59  (1.46 to 1.73) | 1.15  (1.10 to 1.21) | 0.69  (0.68 to 0.70) | 0.20  (0.18 to 0.21) | 0.33  (0.29 to 0.38) | 0.22  (0.22 to 0.22) |
| Digestive diseases | 3.50  (3.46 to 3.54) | 5.03  (4.96 to 5.10) | 4.32  (4.06 to 4.60) | 6.57  (6.27 to 6.87) | 5.16  (4.65 to 5.71) | 4.82  (4.79 to 4.85) | 6.84  (6.47 to 7.22) |
| Musculoskeletal disorders | 0.10  (0.10 to 0.10) | 0.05  (0.04 to 0.06) | 0.19  (0.16 to 0.23) | 0.14  (0.12 to 0.15) | 0.06  (0.06 to 0.06) | 0.23  (0.22 to 0.25) | 0.08  (0.08 to 0.08) |
| Skin and subcutaneous diseases | 0.11  (0.10 to 0.13) | 0.07  (0.03 to 0.13) | 0.23  (0.12 to 0.40) | 0.35  (0.21 to 0.55) | 0.08  (0.06 to 0.11) | 0.19  (0.14 to 0.25) | 0.15  (0.14 to 0.16) |
| Other non-communicable diseases | 0.89  (0.82 to 0.96) | 0.89  (0.75 to 1.04) | 1.63  (1.50 to 1.77) | 2.15  (1.91 to 2.42) | 1.15  (1.04 to 1.27) | 1.28  (1.18 to 1.38) | 1.75  (1.42 to 2.13) |
| **Communicable, maternal, neonatal, and nutritional diseases** | | | | | | | |
| Neglected tropical diseases and malaria | 0.08  (0.05 to 0.14) | 0.01  (0.00 to 0.01) | 0.01  (0.00 to 0.03) | 0.34  (0.19 to 0.57) | 0.17  (0.06 to 0.38) | 0.35  (0.21 to 0.57) | 4.89  (2.88 to 7.81) |
| HIV/AIDS and sexually transmitted infections | 0.38  (0.27 to 0.52) | 0.59  (0.54 to 0.66) | 0.14  (0.12 to 0.15) | 0.74  (0.68 to 0.80) | 0.14  (0.06 to 0.30) | 0.28  (0.15 to 0.48) | 5.61  (4.92 to 6.35) |
| Respiratory infections and tuberculosis | 4.32  (4.26 to 4.39) | 2.94  (2.87 to 3.01) | 5.21  (5.08 to 5.33) | 6.31  (6.01 to 6.63) | 3.64  (3.62 to 3.66) | 8.60  (8.35 to 8.85) | 15.63  (14.20 to 17.18) |
| Enteric infections | 0.64  (0.41 to 0.96) | 0.06  (0.04 to 0.08) | 0.30  (0.30 to 0.30) | 0.59  (0.53 to 0.65) | 0.48  (0.30 to 0.73) | 5.80  (3.70 to 8.72) | 6.04  (4.37 to 8.11) |
| Other infectious diseases | 0.36  (0.30 to 0.43) | 0.18  (0.15 to 0.20) | 0.18  (0.15 to 0.21) | 0.28  (0.25 to 0.32) | 0.60  (0.44 to 0.79) | 1.20  (1.09 to 1.31) | 2.00  (1.85 to 2.15) |
| Maternal and neonatal disorders | 0.61  (0.57 to 0.66) | 0.49  (0.44 to 0.54) | 0.23  (0.23 to 0.24) | 0.89  (0.83 to 0.97) | 1.01  (0.99 to 1.04) | 2.31  (2.30 to 2.32) | 2.41  (2.40 to 2.42) |
| Nutritional deficiencies | 0.31  (0.29 to 0.32) | 0.03  (0.02 to 0.03) | 0.17  (0.17 to 0.17) | 0.78  (0.74 to 0.81) | 0.15  (0.14 to 0.17) | 0.21  (0.20 to 0.22) | 0.87  (0.87 to 0.87) |
| **Injuries** | | | | | | | |
| Transport injuries | 2.15  (1.89 to 2.43) | 1.49  (1.40 to 1.58) | 1.12  (1.05 to 1.19) | 2.58  (2.55 to 2.61) | 3.52  (3.01 to 4.09) | 1.95  (1.68 to 2.26) | 2.50  (2.40 to 2.61) |
| Self-harm and interpersonal violence | 1.10  (0.94 to 1.28) | 3.28  (3.07 to 3.49) | 1.99  (1.85 to 2.13) | 4.07  (3.96 to 4.19) | 1.82  (1.77 to 1.88) | 1.55  (1.37 to 1.73) | 2.42  (2.37 to 2.47) |
| Unintentional injuries | 2.58  (2.25 to 2.94) | 3.23  (3.17 to 3.30) | 2.78  (2.65 to 2.91) | 2.86  (2.74 to 2.98) | 2.06  (1.89 to 2.25) | 3.90  (3.85 to 3.95) | 2.72  (2.71 to 2.72) |

Source: Global Burden of Disease (GBD/IHME). Note: 95% uncertainty interval in parenthesis. Central Europe, Eastern Europe, and Central Asia (CEC); High-income (HI); Latin America and the Caribbean (LAC); North Africa and Middle East (MENA); South Asia (SA); Southeast Asia, East Asia, and Oceania (SEO); and Sub-Saharan Africa (SSA).

## **Fracionalization index without and with age structure correction**

In this paper, the fractionalization indicator was calculated from the proportion of deaths by causes of deaths from the life tables. The COD profile is associated with the age structure of deaths in a population. Therefore, it is necessary to correct the COD diversity indicator for the population's age structure to compare different populations at different points in time. In Table A6, we can observe the Fractionalization indicators values without and with age structure correction. The fractionalization indicator is generally higher when not controlling for age structure but presenting the same temporal trajectory to the indicator corrected for age structure.

### Table A6 - Fracionalization index without and with age structure correction – regions and sex (1990 and 2019)

|  | **1990** | | **2019** | |
| --- | --- | --- | --- | --- |
| **Females** | **F without age structure correction** | **F with age structure correction** | **F without age structure correction** | **F with age structure correction** |
| SEO | 0.858 (0.827 to 0.889) | 0.795 (0.771 to 0.820) | 0.759 (0.715 to 0.806) | 0.732 (0.695 to 0.771) |
| CEC | 0.595 (0.560 to 0.632) | 0.551 (0.518 to 0.587) | 0.588 (0.534 to 0.647) | 0.563 (0.517 to 0.614) |
| HI | 0.729 (0.686 to 0.773) | 0.712 (0.687 to 0.739) | 0.794 (0.727 to 0.866) | 0.791 (0.776 to 0.807) |
| LAC | 0.880 (0.865 to 0.895) | 0.801 (0.782 to 0.820) | 0.847 (0.817 to 0.877) | 0.827 (0.812 to 0.842) |
| MENA | 0.864 (0.835 to 0.895) | 0.712 (0.662 to 0.766) | 0.772 (0.737 to 0.808) | 0.685 (0.640 to 0.732) |
| AS | 0.894 (0.844 to 0.944) | 0.872 (0.871 to 0.872) | 0.876 (0.833 to 0.920) | 0.849 (0.832 to 0.866) |
| SSA | 0.899 (0.843 to 0.960) | 0.888 (0.875 to 0.901) | 0.909 (0.874 to 0.946) | 0.867 (0.844 to 0.890) |
| **Males** |  |  |  |  |
| SEO | 0.874 (0.853 to 0.895) | 0.819 (0.800 to 0.838) | 0.777 (0.756 to 0.799) | 0.757 (0.734 to 0.780) |
| CEC | 0.749 (0.739 to 0.758) | 0.702 (0.689 to 0.715) | 0.709 (0.689 to 0.729) | 0.682 (0.655 to 0.708) |
| HI | 0.765 (0.750 to 0.780) | 0.748 (0.735 to 0.761) | 0.794 (0.764 to 0.825) | 0.796 (0.781 to 0.811) |
| LAC | 0.902 (0.892 to 0.911) | 0.840 (0.829 to 0.852) | 0.874 (0.857 to 0.891) | 0.848 (0.835 to 0.860) |
| MENA | 0.874 (0.847 to 0.901) | 0.756 (0.715 to 0.798) | 0.799 (0.770 to 0.829) | 0.729 (0.694 to 0.767) |
| SA | 0.901 (0.868 to 0.934) | 0.875 (0.866 to 0.884) | 0.869 (0.847 to 0.892) | 0.839 (0.819 to 0.860) |
| SSA | 0.901 (0.854 to 0.949) | 0.897 (0.886 to 0.908) | 0.916 (0.886 to 0.947) | 0.892 (0.877 to 0.907) |

Source: Global Burden of Disease (GBD/IHME). Note: Central Europe, Eastern Europe, and Central Asia (CEC); High-income (HI); Latin America and the Caribbean (LAC); North Africa and Middle East (MENA); South Asia (SA); Southeast Asia, East Asia, and Oceania (SEO); and Sub-Saharan Africa (SSA).

## **Uncertainty interval for contribution for cause for fractionalization index variation in the Horiuchi decomposition**

Tables A6 to A7 present the uncertainty intervals for the contribution for cause for fractionalization index variation in the Horiuchi decomposition, which is presented in tables 2A and 2B of the main text, calculated as described Additional File Section 2.4 and 2.5.

### Table A7 – Decomposition of the fractionalization index variation with uncertainty interval, by region – Females (1990 to 2019)

|  | **SEO** | **CEC** | **HI** | **LAC** | **MENA** | **SA** | **SSA** |
| --- | --- | --- | --- | --- | --- | --- | --- |
| **Non-communicable diseases** | | | | | | | |
| Cardiovascular diseases | -0.082 | 0.016 | 0.103 | 0.042 | -0.021 | -0.045 | -0.034 |
|  | (-0.099 to -0.065) | (-0.001 to 0.034) | (0.097 to 0.109) | (0.042 to 0.042) | (-0.026 to -0.016) | (-0.054 to -0.035) | (-0.04 to -0.029) |
| Neoplasms | -0.007 | -0.003 | -0.011 | -0.005 | -0.005 | -0.006 | -0.005 |
|  | (-0.007 to -0.007) | (-0.004 to -0.002) | (-0.017 to -0.005) | (-0.006 to -0.004) | (-0.006 to -0.005) | (-0.006 to -0.005) | (-0.006 to -0.005) |
| Diabetes and kidney diseases | -0.002 | 0.000 | -0.002 | -0.007 | -0.003 | -0.003 | -0.003 |
|  | (-0.002 to -0.001) | (0.000 to 0.000) | (-0.002 to -0.001) | (-0.008 to -0.006) | (-0.004 to -0.003) | (-0.003 to -0.002) | (-0.003 to -0.002) |
| Chronic respiratory diseases | 0.025 | 0.001 | -0.002 | -0.001 | 0.000 | -0.008 | 0.000 |
|  | (0.023 to 0.028) | (0.000 to 0.001) | (-0.002 to -0.001) | (-0.001 to -0.001) | (0.000 to 0.000) | (-0.009 to -0.008) | (0.000 to 0.000) |
| Mental disorders | 0.000 | 0.000 | 0.000 | 0.000 | 0.000 | 0.000 | 0.000 |
|  | (0.000 to 0.000) | (0.000 to 0.000) | (0.000 to 0.000) | (0.000 to 0.000) | (0.000 to 0.000) | (0.000 to 0.000) | (0.000 to 0.000) |
| Neurological disorders | -0.004 | -0.002 | -0.009 | -0.005 | -0.002 | -0.001 | -0.001 |
|  | (-0.01 to 0.003) | (-0.005 to 0.001) | (-0.021 to 0.002) | (-0.011 to 0.002) | (-0.005 to 0.001) | (-0.003 to 0.000) | (-0.002 to 0.000) |
| Substance use disorders | 0.000 | 0.000 | 0.000 | 0.000 | 0.000 | 0.000 | 0.000 |
|  | (0.000 to 0.000) | (0.000 to 0.000) | (0.000 to 0.000) | (0.000 to 0.000) | (0.000 to 0.000) | (0.000 to 0.000) | (0.000 to 0.000) |
| Digestive diseases | 0.001 | -0.001 | 0.000 | 0.000 | 0.001 | 0.000 | 0.000 |
|  | (0.001 to 0.001) | (-0.001 to -0.001) | (0.000 to 0.000) | (0.000 to 0.000) | (0.001 to 0.001) | (0.000 to 0.000) | (0.000 to 0.000) |
| Musculoskeletal disorders | 0.000 | 0.000 | 0.000 | 0.000 | 0.000 | 0.000 | 0.000 |
|  | (0.000 to 0.000) | (0.000 to 0.000) | (0.000 to 0.000) | (0.000 to 0.000) | (0.000 to 0.000) | (0.000 to 0.000) | (0.000 to 0.000) |
| Skin and subcutaneous diseases | 0.000 | 0.000 | 0.000 | 0.000 | 0.000 | 0.000 | 0.000 |
|  | (0.000 to 0.000) | (0.000 to 0.000) | (0.000 to 0.000) | (0.000 to 0.000) | (0.000 to 0.000) | (0.000 to 0.000) | (0.000 to 0.000) |
| Other non-communicable diseases | 0.000 | 0.000 | 0.000 | 0.000 | 0.000 | 0.000 | 0.000 |
|  | (0.000 to 0.000) | (0.000 to 0.000) | (0.000 to 0.000) | (-0.001 to 0.000) | (0.000 to 0.000) | (0.000 to 0.000) | (0.000 to 0.000) |
| **Communicable, maternal, neonatal, and nutritional diseases** | | | | | | | |
| Neglected tropical diseases and malaria | 0.000 | 0.000 | 0.000 | 0.000 | 0.000 | 0.000 | 0.002 |
|  | (0.000 to 0.000) | (0.000 to 0.000) | (0.000 to 0.000) | (0.000 to 0.000) | (0.000 to 0.000) | (0.000 to 0.000) | (0.002 to 0.002) |
| HIV/AIDS and sexually transmitted infections | 0.000 | 0.000 | 0.000 | 0.000 | 0.000 | 0.000 | -0.002 |
|  | (0.000 to 0.000) | (0.000 to 0.000) | (0.000 to 0.000) | (0.000 to 0.000) | (0.000 to 0.000) | (0.000 to 0.000) | (-0.002 to -0.002) |
| Respiratory infections and tuberculosis | 0.004 | 0.000 | 0.000 | 0.001 | 0.001 | 0.009 | 0.009 |
|  | (0.004 to 0.004) | (0.000 to 0.000) | (0.000 to 0.000) | (0.001 to 0.001) | (0.001 to 0.002) | (0.007 to 0.01) | (0.007 to 0.011) |
| Enteric infections | 0.000 | 0.000 | 0.000 | 0.001 | 0.000 | 0.028 | 0.011 |
|  | (0.000 to 0.001) | (0.000 to 0.000) | (0.000 to 0.000) | (0.000 to 0.001) | (0.000 to 0.000) | (0.018 to 0.037) | (0.004 to 0.017) |
| Other infectious diseases | 0.000 | 0.000 | 0.000 | 0.000 | 0.000 | 0.001 | 0.002 |
|  | (0.000 to 0.000) | (0.000 to 0.000) | (0.000 to 0.000) | (0.000 to 0.000) | (0.000 to 0.001) | (0.001 to 0.001) | (0.001 to 0.002) |
| Maternal and neonatal disorders | 0.000 | 0.000 | 0.000 | 0.000 | 0.001 | 0.002 | 0.001 |
|  | (0.000 to 0.000) | (0.000 to 0.000) | (0.000 to 0.000) | (0.000 to 0.000) | (0.001 to 0.001) | (0.001 to 0.002) | (0.001 to 0.002) |
| Nutritional deficiencies | 0.000 | 0.000 | 0.000 | 0.000 | 0.000 | 0.001 | 0.000 |
|  | (0.000 to 0.000) | (0.000 to 0.000) | (0.000 to 0.000) | (0.000 to 0.000) | (0.000 to 0.000) | (0.000 to 0.001) | (0.000 to 0.000) |
| **Injuries** | | | | | | | |
| Transport injuries | 0.000 | 0.000 | 0.000 | 0.000 | 0.000 | 0.000 | 0.000 |
|  | (0.000 to 0.000) | (0.000 to 0.000) | (0.000 to 0.000) | (0.000 to 0.000) | (0.000 to 0.000) | (0.000 to 0.000) | (0.000 to 0.000) |
| Self-harm and interpersonal violence | 0.000 | 0.000 | 0.000 | 0.000 | 0.000 | 0.000 | 0.000 |
|  | (0.000 to 0.000) | (0.000 to 0.000) | (0.000 to 0.000) | (0.000 to 0.000) | (0.000 to 0.000) | (0.000 to 0.000) | (0.000 to 0.000) |
| Unintentional injuries | 0.000 | 0.000 | 0.000 | 0.000 | 0.000 | -0.001 | 0.000 |
|  | (0.000 to 0.000) | (0.000 to 0.000) | (0.000 to 0.000) | (0.000 to 0.000) | (0.000 to 0.000) | (-0.001 to -0.001) | (0.000 to 0.000) |

Source: Global Burden of Disease (GBD/IHME). Note: 95% uncertainty interval in parenthesis. Central Europe, Eastern Europe, and Central Asia (CEC); High-income (HI); Latin America and the Caribbean (LAC); North Africa and Middle East (MENA); South Asia (SA); Southeast Asia, East Asia, and Oceania (SEO); and Sub-Saharan Africa (SSA).

### Table A8 - Decomposition of the fractionalization index variation with uncertainty interval, by region – Males (1990 to 2019)

|  | **SEO** | **CEC** | **HI** | **LAC** | **MENA** | **SA** | **SSA** |
| --- | --- | --- | --- | --- | --- | --- | --- |
| **Non-communicable diseases** | | | | | | | |
| Cardiovascular diseases | -0.068 | -0.021 | 0.071 | 0.024 | -0.019 | -0.049 | -0.020 |
|  | (-0.074 to -0.063) | (-0.036 to -0.006) | (0.07 to 0.071) | (0.023 to 0.025) | (-0.025 to -0.014) | (-0.057 to -0.042) | (-0.022 to -0.017) |
| Neoplasms | -0.018 | -0.001 | -0.016 | -0.010 | -0.008 | -0.005 | -0.006 |
|  | (-0.02 to -0.017) | (-0.001 to -0.001) | (-0.022 to -0.009) | (-0.012 to -0.008) | (-0.01 to -0.007) | (-0.005 to -0.005) | (-0.007 to -0.005) |
| Diabetes and kidney diseases | -0.001 | 0.000 | -0.002 | -0.008 | -0.002 | -0.003 | -0.002 |
|  | (-0.001 to -0.001) | (0.000 to 0.000) | (-0.002 to -0.002) | (-0.008 to -0.007) | (-0.002 to -0.002) | (-0.003 to -0.002) | (-0.003 to -0.002) |
| Chronic respiratory diseases | 0.019 | 0.002 | -0.001 | -0.001 | 0.000 | -0.005 | 0.000 |
|  | (0.018 to 0.02) | (0.002 to 0.002) | (-0.001 to -0.001) | (-0.001 to 0.000) | (0.000 to 0.000) | (-0.005 to -0.005) | (0.000 to 0.000) |
| Mental disorders | 0.000 | 0.000 | 0.000 | 0.000 | 0.000 | 0.000 | 0.000 |
|  | (0.000 to 0.000) | (0.000 to 0.000) | (0.000 to 0.000) | (0.000 to 0.000) | (0.000 to 0.000) | (0.000 to 0.000) | (0.000 to 0.000) |
| Neurological disorders | -0.001 | -0.001 | -0.004 | -0.002 | -0.001 | -0.001 | 0.000 |
|  | (-0.002 to 0.000) | (-0.001 to 0.000) | (-0.01 to 0.002) | (-0.006 to 0.001) | (-0.003 to 0.001) | (-0.002 to 0.000) | (-0.001 to 0.000) |
| Substance use disorders | 0.000 | 0.000 | 0.000 | 0.000 | 0.000 | 0.000 | 0.000 |
|  | (0.000 to 0.000) | (0.000 to 0.000) | (0.000 to 0.000) | (0.000 to 0.000) | (0.000 to 0.000) | (0.000 to 0.000) | (0.000 to 0.000) |
| Digestive diseases | 0.001 | -0.001 | 0.000 | 0.000 | 0.000 | 0.001 | -0.001 |
|  | (0.001 to 0.001) | (-0.001 to -0.001) | (0.000 to 0.000) | (0.000 to 0.000) | (0.000 to 0.001) | (0.000 to 0.001) | (-0.001 to -0.001) |
| Musculoskeletal disorders | 0.000 | 0.000 | 0.000 | 0.000 | 0.000 | 0.000 | 0.000 |
|  | (0.000 to 0.000) | (0.000 to 0.000) | (0.000 to 0.000) | (0.000 to 0.000) | (0.000 to 0.000) | (0.000 to 0.000) | (0.000 to 0.000) |
| Skin and subcutaneous diseases | 0.000 | 0.000 | 0.000 | 0.000 | 0.000 | 0.000 | 0.000 |
|  | (0.000 to 0.000) | (0.000 to 0.000) | (0.000 to 0.000) | (0.000 to 0.000) | (0.000 to 0.000) | (0.000 to 0.000) | (0.000 to 0.000) |
| Other non-communicable diseases | 0.000 | 0.000 | 0.000 | 0.000 | 0.000 | 0.000 | 0.000 |
|  | (0.000 to 0.000) | (0.000 to 0.000) | (0.000 to 0.000) | (0.000 to 0.000) | (0.000 to 0.000) | (0.000 to 0.000) | (0.000 to 0.000) |
| **Communicable, maternal, neonatal, and nutritional diseases** | | | | | | | |
| Neglected tropical diseases and malaria | 0.000 | 0.000 | 0.000 | 0.000 | 0.000 | 0.000 | 0.002 |
|  | (0.000 to 0.000) | (0.000 to 0.000) | (0.000 to 0.000) | (0.000 to 0.000) | (0.000 to 0.000) | (0.000 to 0.000) | (0.002 to 0.002) |
| HIV/AIDS and sexually transmitted infections | 0.000 | 0.000 | 0.000 | 0.000 | 0.000 | 0.000 | -0.002 |
|  | (0.000 to 0.000) | (0.000 to 0.000) | (0.000 to 0.000) | (0.000 to 0.000) | (0.000 to 0.000) | (0.000 to 0.000) | (-0.002 to -0.002) |
| Respiratory infections and tuberculosis | 0.005 | 0.000 | -0.001 | 0.002 | 0.001 | 0.014 | 0.015 |
|  | (0.005 to 0.005) | (0.000 to 0.000) | (-0.001 to 0.000) | (0.002 to 0.002) | (0.001 to 0.001) | (0.012 to 0.016) | (0.011 to 0.019) |
| Enteric infections | 0.000 | 0.000 | 0.000 | 0.001 | 0.000 | 0.010 | 0.007 |
|  | (0.000 to 0.001) | (0.000 to 0.000) | (0.000 to 0.000) | (0.000 to 0.001) | (0.000 to 0.000) | (0.003 to 0.018) | (0.003 to 0.011) |
| Other infectious diseases | 0.000 | 0.000 | 0.000 | 0.000 | 0.000 | 0.001 | 0.002 |
|  | (0.000 to 0.000) | (0.000 to 0.000) | (0.000 to 0.000) | (0.000 to 0.000) | (0.000 to 0.001) | (0.001 to 0.001) | (0.001 to 0.003) |
| Maternal and neonatal disorders | 0.000 | 0.000 | 0.000 | 0.000 | 0.001 | 0.001 | 0.000 |
|  | (0.000 to 0.000) | (0.000 to 0.000) | (0.000 to 0.000) | (0.000 to 0.000) | (0.001 to 0.001) | (0.001 to 0.001) | (0.000 to 0.001) |
| Nutritional deficiencies | 0.000 | 0.000 | 0.000 | 0.000 | 0.000 | 0.000 | 0.000 |
|  | (0.000 to 0.000) | (0.000 to 0.000) | (0.000 to 0.000) | (0.000 to 0.000) | (0.000 to 0.000) | (0.000 to 0.000) | (0.000 to 0.000) |
| **Injuries** | | | | | | | |
| Transport injuries | 0.000 | 0.000 | 0.000 | 0.001 | 0.001 | 0.000 | 0.000 |
|  | (0.000 to 0.000) | (0.000 to 0.000) | (0.000 to 0.000) | (0.000 to 0.001) | (0.001 to 0.001) | (0.000 to 0.000) | (0.000 to 0.000) |
| Self-harm and interpersonal violence | 0.000 | 0.000 | 0.000 | 0.000 | 0.000 | 0.000 | 0.001 |
|  | (0.000 to 0.000) | (0.000 to 0.001) | (0.000 to 0.000) | (0.000 to 0.000) | (0.000 to 0.000) | (0.000 to 0.000) | (0.000 to 0.001) |
| Unintentional injuries | 0.000 | 0.001 | 0.000 | 0.000 | 0.001 | 0.000 | 0.000 |
|  | (0.000 to 0.000) | (0.001 to 0.001) | (0.000 to 0.000) | (0.000 to 0.001) | (0.001 to 0.001) | (0.000 to 0.000) | (0.000 to 0.000) |

Source: Global Burden of Disease (GBD/IHME). Note: 95% uncertainty interval in parenthesis. Central Europe, Eastern Europe, and Central Asia (CEC); High-income (HI); Latin America and the Caribbean (LAC); North Africa and Middle East (MENA); South Asia (SA); Southeast Asia, East Asia, and Oceania (SEO); and Sub-Saharan Africa (SSA).

## **Comparison with results from Bergeron et al. (2020)**

Figure A1 presents the Shannon and Fractionalization indicators for the 15 high-income countries analyzed by Bergeron et al. [11] between 1994 and 2017, using the GBD Project's cause group classification. Overall, the correlation between the two indicators is high (Pearson Correlation of 0.973) and the trajectories over time are very similar, thus indicating the strong correspondence between both indicators. Moreover, it is important to note that the results are very similar to those found by Bergeron et al. [11], thus evidencing the robustness of the analysis.

### Figure A1 - Shannon and Fractionalization indicators – High income countries (1994 to 2017)


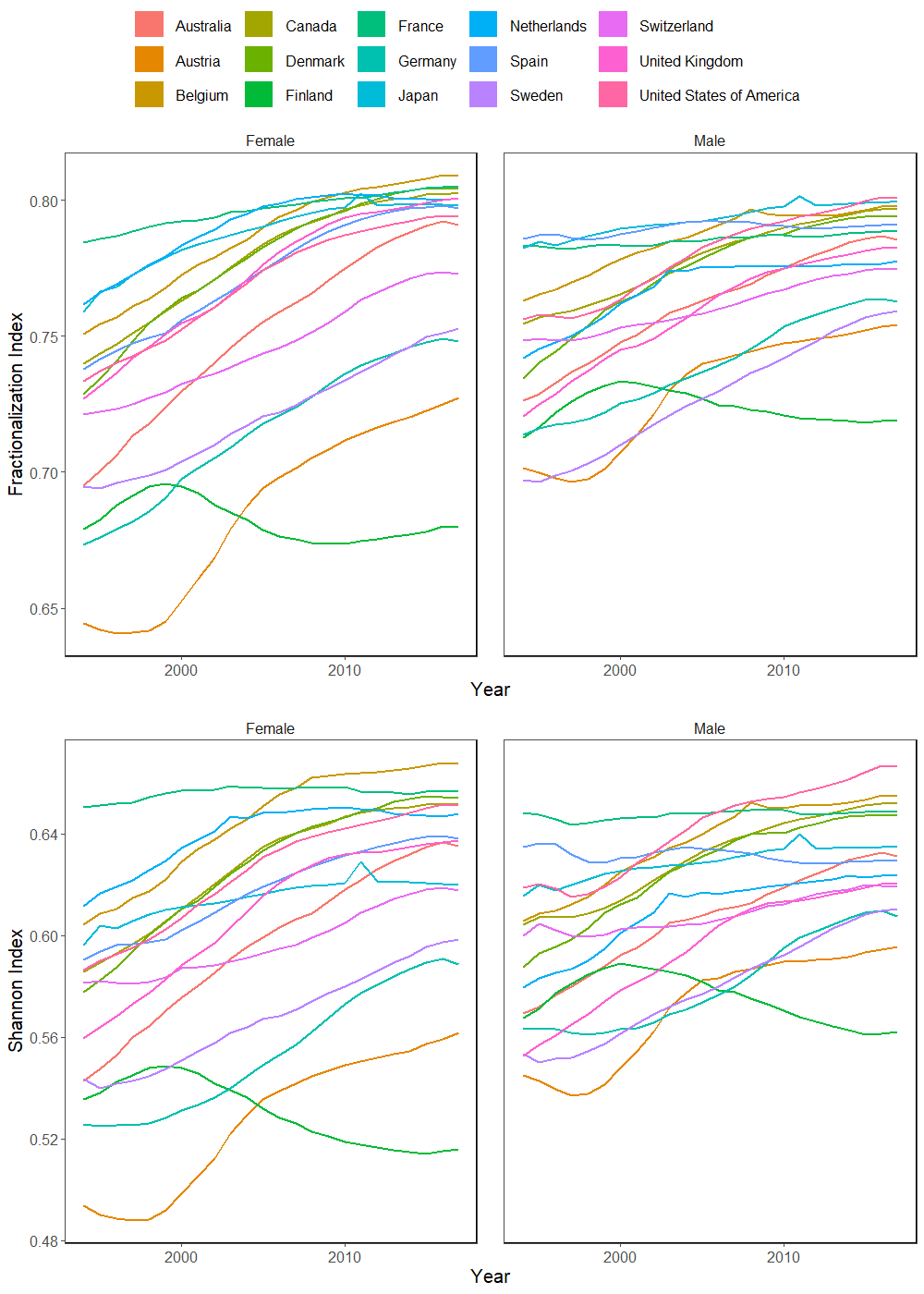


Source: Global Burden of Disease (GBD/IHME). Note: Central Europe, Eastern Europe, and Central Asia (CEC); High-income (HI); Latin America and the Caribbean (LAC); North Africa and Middle East (MENA); South Asia (SA); Southeast Asia, East Asia, and Oceania (SEO); and Sub-Saharan Africa (SSA).

## **Sensitivity analysis**

One of the major limitations to the use of diversity indicators is that they are very sensitive to the number of categories (causes of death) used. Thus, we calculated the fractionalization index using two classification levels of causes of death provided by the GBD project: level 2, with 21 cause groups, and level 3, with 133 cause groups (see Table A9). Although the magnitude between the two estimates is very different, the correlation between them is the very strong and the trends remain relatively constant.

### Table A9 - Fractionalization index by GBD aggregation level of the cause groups – regions and sex (1990 and 2019)

|  | **1990** | | **2019** | |
| --- | --- | --- | --- | --- |
|  | **Level 2**  **(21 groups)** | **Level 3**  **(133 groups)** | **Level 2**  **(21 groups)** | **Level 3**  **(133 groups)** |
| **Females** |  |  |  |  |
| SEO | 0.795 | 0.907 | 0.732 | 0.897 |
| CEC | 0.551 | 0.805 | 0.563 | 0.815 |
| HI | 0.712 | 0.899 | 0.791 | 0.940 |
| LAC | 0.801 | 0.927 | 0.827 | 0.938 |
| NME | 0.712 | 0.879 | 0.685 | 0.871 |
| SA | 0.872 | 0.928 | 0.849 | 0.933 |
| SSA | 0.888 | 0.949 | 0.867 | 0.949 |
| **Correlation with level 2** |  | **0.963*** |  | **0.9717*** |
| **Males** |  |  |  |  |
| SEO | 0.819 | 0.919 | 0.757 | 0.908 |
| CEC | 0.702 | 0.870 | 0.682 | 0.870 |
| HI | 0.748 | 0.910 | 0.796 | 0.945 |
| LAC | 0.840 | 0.938 | 0.848 | 0.944 |
| NME | 0.756 | 0.883 | 0.729 | 0.879 |
| SA | 0.875 | 0.937 | 0.839 | 0.924 |
| SSA | 0.897 | 0.950 | 0.892 | 0.954 |
| **Correlation with level 2** |  | **0.9358*** |  | **0.9228*** |

Source: Global Burden of Disease (GBD/IHME). Note: Central Europe, Eastern Europe, and Central Asia (CEC); High-income (HI); Latin America and the Caribbean (LAC); North Africa and Middle East (MENA); South Asia (SA); Southeast Asia, East Asia, and Oceania (SEO); and Sub-Saharan Africa (SSA).
